# Tight-packing of large pilin subunits provides distinct structural and mechanical properties for the *Myxococcus xanthus* type IVa pilus

**DOI:** 10.1073/pnas.2321989121

**Published:** 2024-04-16

**Authors:** Anke Treuner-Lange, Weili Zheng, Albertus Viljoen, Steffi Lindow, Marco Herfurth, Yves F. Dufrêne, Lotte Søgaard-Andersen, Edward H. Egelman

**Affiliations:** ^a^Department of Ecophysiology, Max Planck Institute for Terrestrial Microbiology, Marburg 35043, Germany; ^b^Department of Biochemistry and Molecular Genetics, University of Virginia School of Medicine, Charlottesville, VA 22903; ^c^Louvain Institute of Biomolecular Science and Technology, UCLouvain, Louvain-la-Neuve B-1348, Belgium

**Keywords:** Type IVa pili, large pilins, cryo-EM, intersubunit contacts, bacterial motility

## Abstract

Type IVa pili (T4aP) are ubiquitous cell surface filaments important for surface motility, adhesion to surfaces, DNA uptake, biofilm formation, and virulence. T4aP are built from thousands of copies of the major pilin subunit and tipped by a complex composed of minor pilins and in some systems also the PilY1 adhesin. While major pilins of structurally characterized T4aP have lengths of <165 residues, the major pilin PilA of *Myxococcus xanthus* is unusually large with 208 residues. All major pilins have a conserved N-terminal domain and a variable C-terminal domain, and the additional residues of PilA are due to a larger C-terminal domain. We solved the structure of the *M. xanthus* T4aP (T4aP^Mx^) at a resolution of 3.0 Å using cryo-EM. The T4aP^Mx^ follows the structural blueprint of other T4aP with the pilus core comprised of the interacting N-terminal α1-helices, while the globular domains decorate the T4aP surface. The atomic model of PilA built into this map shows that the large C-terminal domain has more extensive intersubunit contacts than major pilins in other T4aP. As expected from these greater contacts, the bending and axial stiffness of the T4aP^Mx^ is significantly higher than that of other T4aP and supports T4aP-dependent motility on surfaces of different stiffnesses. Notably, T4aP^Mx^ variants with interrupted intersubunit interfaces had decreased bending stiffness, pilus length, and strongly reduced motility. These observations support an evolutionary scenario whereby the large major pilin enables the formation of a rigid T4aP that expands the environmental conditions in which the T4aP system functions.

## Significance

To translocate on solid surfaces many bacteria use dynamic cell surface structures called type IVa pili (T4aP). T4aP undergo cycles of extension, adhesion to a surface, and retraction that generate a force sufficient to pull a cell forward. The T4aP filament is composed of thousands of copies of the major pilin. Here, using cryo-EM, we solve the structure of the T4aP of *Myxococcus xanthus* (T4aP^Mx^), which has an unusual large major pilin called PilA. The large major pilins of T4aP^Mx^ are tightly packed and have extensive intersubunit contacts, making the T4aP^Mx^ more rigid and stronger than other studied T4aP. Interestingly, large major T4a pilins are found in several bacterial phyla and might represent an evolutionary adaptation to specific environments.

Bacterial motility is important for virulence, colonization of various habitats, biofilm formation, interactions with host cells, and fitness by directing cells toward nutrients and away from toxins and predators (1). Accordingly, bacteria can move in many different environments and their motility devices are adapted to these varying conditions (1, 2). Generally, bacteria move using highly conserved nanomachines that energize either the rotation of flagella to enable swimming in liquids and swarming on semisolid surfaces or the extension/retraction of type IVa pili (T4aP) to enable translocation on solid surfaces (1, 2). Some bacteria also move on surfaces by gliding, but the involved nanomachines are more diverse, each with a narrow taxonomic distribution (1, 2). Here, we focus on T4aP, which are not only important for motility but also for adhesion to host cells and abiotic surfaces, natural transformation with horizontal gene transfer, biofilm formation, virulence, predation, and surface sensing (3, 4).

T4aP are part of the large superfamily of type 4 filaments (T4F), among which T4aP, T4bP, T4cP (formerly Tad/Flp), endopili of the type II secretion system (previously called pseudopili), Msh pili, and Com pili are present in bacteria (5, 6). All these T4F are composed of major and minor pilins; however, motility is exclusively mediated by T4aP (5, 6). The versatility of T4aP depends on their ability to undergo cycles of extension with adhesion to a surface, and retraction that generates a force sufficient to pull a cell forward (7–10). These cycles are driven by the T4aP machine (T4aPM), which is composed of ~15 conserved proteins forming a large macromolecular complex that spans from the outer membrane across the periplasm and inner membrane (IM) to the cytoplasm (11–13). T4aP extension and retraction are powered by the ATPases PilB and PilT, respectively that bind to the cytoplasmic base of the T4aPM in a mutually exclusive manner (3, 12, 14). All ~15 proteins are essential for T4aP extension except for PilT, which is only necessary for retraction (3). The T4aP are flexible, thin, up to several microns in length, and composed of thousands of copies of the major pilin subunit as well as a tip complex comprising minor pilins and sometimes also the PilY1 adhesin (13, 15–19). During T4aP extension, major pilin subunits are extracted from a reservoir in the IM and inserted at the base of the growing pilus; during T4aP retraction, this process is reversed, and the major pilin subunits are removed from the base of the T4aP and reinserted into the IM (3).

Major pilins are synthesized as prepilins with an N-terminal type III signal peptide (SPIII), which is cleaved off by the PilD prepilin peptidase between the Gly and Phe residues in the consensus GFxxxE motif to generate the mature major pilin (hereafter simply referred to as the major pilin) (20). A variety of additional posttranslational modifications, such as methylation, glycosylation, and modifications with phosphate or phospholipids are reported for T4a pilins (4). Most major pilins have one conserved C-terminal disulfide-bonded loop (D-region) (4); however pilins with two disulfide bonds (21) or lacking cysteine residues completely have also been reported (22). Sequence and structural analyses of major pilins in isolation and structural studies of intact T4aP filaments have shown that major pilins share the same overall structure with a semiconserved N-terminal α-helix (α1) and a highly variable C-terminal, largely β-stranded, globular domain (4, 19). The α1-helix can be divided into the mainly hydrophobic highly conserved N-terminal part (α1-N), which is essential for anchoring the pilin in the IM before its incorporation into the pilus, and the less conserved amphipathic C-terminal part (α1-C) that connects to and packs against the globular C-terminal domain (4, 19).

The structures of eight bacterial T4aP have been solved to a resolution of 3.2 to 8.0 Å, from *Neisseria gonorrhoeae* (PDB 5VXX) (23), *N. meningitidis* (PDB 5KUA) (24)*, Pseudomonas aeruginosa* (PDB 5VXY) (23)*, Escherichia coli* (PDB 6GV9) (21), two different ones from *Thermus thermophilus* (PDB 6XXD and 6XXE) (25), *Geobacter sulfurreducens* (PDB 6VK9 and 7TGG) (26, 27), and *Streptococcus sanguinis* (PDB 8PFB) (17). With the exception of the *G. sulfurreducens* and *S. sanguinis* structures, these T4aP are composed of thousands of copies of the same major pilin; by contrast, the *G. sulfurreducens* T4aP is composed of a major pilin in which the two domains are encoded by two different genes resulting in a pilin subunit containing two polypeptide chains (26, 28), and the T4aP of *S. sanguinis* is heteropolymeric and composed of two very similar major T4a pilins, PilE1 and PilE2 (17, 29).

These structures revealed, not surprisingly, that all T4aP filaments share the same overall architecture. Specifically, the major pilins are helically arranged and tightly packed, giving rise to pili with widths of ~60 to 75 Å, a rise of ~9 to 11 Å per subunit, and ~4 subunits per turn, with ~1,000 subunits per micron length of the T4aP. The pilus core comprises the extensively interacting α1-helices while the variable globular domains decorate the T4aP surface. In this conserved structural blueprint, the α1-helices establish the backbone of the T4aP, while the divergent globular domains determine the shape, surface charge, and functional properties of T4aP (17, 21, 23–26, 30). While the C-terminal globular domain remains largely unchanged upon the incorporation of a major pilin into the pilus, the α1-helix undergoes a partial loss of α-helical structure of variable length around the highly conserved Pro22 residue (17, 23–26). It was suggested that the melting of this segment is essential for the tight packing of the major pilins (23). The extensive interactions between major pilins make T4aP highly robust, and in the case of *N. gonorrhoeae* and *M. xanthus,* T4aP were shown to withstand pulling forces of 110 to 150 pN, respectively, during retraction (9, 10). In addition, T4aP of *N. gonorrhoeae, N. meningitidis,* and *P. aeruginosa* have been shown to be highly extensible, undergoing force-induced conformational changes to elongate in response to pulling forces (31–34). Moreover, in *N. gonorrhoeae,* these conformational changes were shown to be reversible (32). Whether this resilience is a conserved feature of T4aP is not known. It was proposed (23) that further melting of the N-terminal α-helix is responsible for the extensibility of these filaments, and the restoring force after extension would be provided by the refolding of the α-helix.

Major T4a pilins are not only diverse in sequence but also in size (4). Among the solved T4aP structures, the major pilins vary in size from 111 to 161 residues (17, 21, 23–25), while the two polypeptide chains forming the *G. sulfurreducens* pilin have a combined size of 165 residues (26, 27). *M. xanthus* is a model system for understanding the architecture and mechanism of the T4aPM (12, 13). Of note, the major pilin PilA of *M. xanthus* contains 208 residues (35) and is, thus, significantly larger than those of solved T4aP structures. Moreover, the *M. xanthus* T4aP (henceforth T4aP^Mx^) is highly robust and can withstand a pulling force of 150 pN during retraction (9). *M. xanthus* is a predatory soil bacterium and belongs to the Myxococcota, prolific secondary metabolite producers (36). *M. xanthus* has a biphasic nutrient-regulated lifestyle in which cells organize to form spreading, predatory colonies in the presence of nutrients and spore-filled fruiting bodies in the absence of nutrients (37, 38). In both phases of the lifestyle, motility has a key function. *M. xanthus* has two motility systems for translocation across surfaces, one for gliding and one that depends on T4aP (37, 38). These two motility systems enable *M. xanthus* cells to translocate on highly diverse surfaces (39).

To understand the properties conferred by large major pilins to T4aP filaments, we determined the structure of T4aP^Mx^ using cryo-EM to a resolution of 3.0 Å, which allowed us to build de novo an atomic model of the entire major pilin and analyzed its biophysical properties in vitro. This structure revealed a T4aP that differed from all existing T4aP structures since it is packed tightly and has much more extensive contacts between the globular domains. Consistent with such a structure, the stiffness of T4aP^Mx^ is significantly higher than that of *N. gonorrhoeae* and *P. aeruginosa* T4aP. Structure-guided mutagenesis of PilA showed that alteration of subunit interfaces caused a reduction in the axial stiffness and length of the T4aP, and these variant T4aP were less efficient in supporting motility on surfaces of different stiffness.

## Results

### Major T4a Pilins Vary Significantly in Size.

**Fig. 1.**
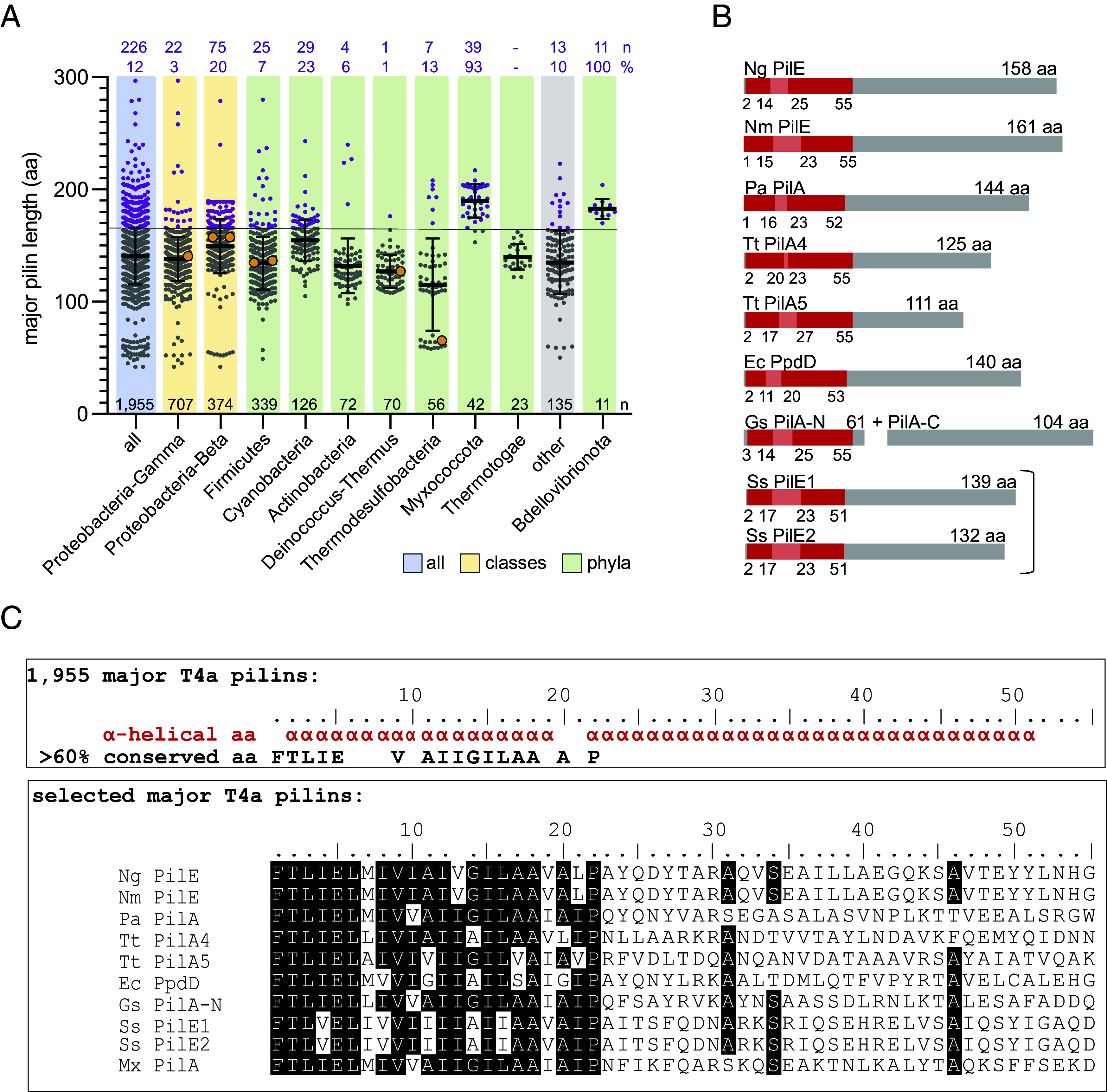
Length distribution of T4a pilins in various phyla/classes. (*A*) Length distribution of major pilins of the KEGG orthology group K02650 (type IV pilus assembly protein PilA) in the complete dataset of 1,955 sequences (light blue, all) grouped according to taxonomy at the phylum (light green) and class level (light yellow); the “other” category (light gray) includes sequences from phyla/classes with <1% of all 1,955 sequences and in which major T4aP pilins are not enriched (*SI Appendix*, Table S2). Gray dots, major pilins ≤165 aa; purple dots, large major pilins (≥166 aa), and orange dots, major pilins of solved T4aP of the K02650 group (PilE from *N. meningitidis* (Nm PilE; 5KUA) (24); PilA from *P. aeruginosa* PAK (Pa PilA; 5VXY) (23); PilE from *N. gonorrhoeae* (Ng PilE; 5VXX) (23); PilA4 from *T. thermophilus* (Tt PilA4; 6XXD) (25); PilA-N from *G. sulfurreducens* (Gs PilA-N; 6VK9) (26) and protein homologs of PilE1 and PilE2 from *S. sanguinis* 2908 (17) (SSA_2314/2315 from *S. sanguinis* SK36). Error bars, mean length ± SD. Black numbers (n) above the *x* axis, total number of sequences in a phylum/class. Purple numbers (n), number of large major pilins, and their % of the total number in a phylum/class. Please note that PpdD from enterohemorrhagic *E. coli* (Ec PpdD; 6GV9) (21) and PilA5 from *T. thermophilus* (Tt PilA5; 6XXE) (25) are not depicted because PpdD belongs to K02682 (prepilin peptidase dependent protein D), which is composed of predominantly enterobacterial PpdD homologous sequences with a full length <150 aa, and PilA5 is not yet assigned to a KEGG group. (*B*) Schematic of overall architecture of major pilins of solved T4aP structures. Red boxes, α1-N and α1-C, with the melted stretch in-between in light red. Numbers, total length of the α1-helix, and the end as well as start of α1-N and α1-C. The sequences used are as in *A*. Note that the major pilin of G. *sulfurreducens* is heterodimeric and composed of PilA-N (61 aa) and PilA-C (104 aa) (26), and the T4aP from *S. sanguinis* is heteropolymeric and composed of two very similar major pilins PilE1 and PilE2 (Ss PilE1 and Ss PilE2; 8PFB) (17). (*C*) Consensus secondary structure (α-helical aa) and amino acid sequence (≥60% conserved aa) for the N termini (1 to 55 aa) in the 1,955 major T4a pilin sequences (upper box). Lower box, sequences of the N termini (1 to 55 aa) of T4a major pilins from solved T4aP structures (as in *A* and *B*) and of PilA from *M. xanthus* (Mx PilA) (13). Residues on black are conserved in ≥60% of the proteins.

To systematically assess the size of major T4a pilins, we first extracted all sequences of the K02650 group (type IV pilus assembly protein PilA) from the KEGG Orthology (KO) database (40). After filtering out sequences with >90% sequence identity, sequences lacking a SPIII, and/or sequences lacking a classified taxonomy, we obtained a set of 1,955 prepilins of T4aP (Dataset S1). After removal of the SPIII, the major pilins vary in length from 42 to 297 aa, with a mean of 141 ± 25 aa, in good agreement with a previous estimate based on fewer sequences (19) (Fig. 1*A*). Because the largest structurally characterized major pilin has a size of 161 aa (and 165 aa for the heterodimeric *G. sulfurreducens* major pilin), we arbitrarily defined large major pilins as proteins with a size ≥166 aa. Among our set of 1,955 sequences, 226 proteins, representing 12%, fulfilled this criterion (Fig. 1*A* and *SI Appendix*, Table S1). These proteins are widespread and present in 13 of the 21 phyla with major pilins, and largely group according to phylogeny (Fig. 1*A* and *SI Appendix*, Fig. S1 and Table S2). However, their distribution in phyla and classes is highly skewed, and at the phyla and class levels, they are overrepresented in Betaproteobacteria (20%), Cyanobacteria (23%), Myxococcota (93%) and Bdellovibrionota (100%) (Fig. 1*A*). Moreover, while the length distribution of major pilins in Betaproteobacteria (42 to 279 aa) and Cyanobacteria (105 to 243 aa) is broad (Fig. 1*A*), it is more narrow in the predatory Myxococcota (153 to 217 aa) and Bdellovibrionota (170 to 204 aa) (Fig. 1*A*). Interestingly, in the Betaproteobacteria, the large major pilins are enriched explicitly in the order Burkholderiales (66 of 75) (*SI Appendix*, Table S1), an ecologically diverse order that includes plant, animal and human pathogens (41), and especially in the *B. cepacia* complex, which is associated with cystic fibrosis (42, 43). Similarly, in the Firmicutes, the large major pilins are highly enriched in the order Eubacteriales (19 of 25), which includes several species of the gut microbiome (*SI Appendix*, Table S1) in which T4P are ubiquitous (44, 45).

In the solved T4aP structures, the major pilins vary not only in size but also in the length of the α1-helix (51 to 55 aa, Fig. 1*B*) and the length of the melted stretch within α1 (2 to 10 aa, Fig. 1*B*) (17, 21, 23–26). To understand the size variation among the 1,955 major T4a pilins, we performed sequence analyses and secondary structure predictions based on multiple sequence and structure alignments of the 1,955 major pilins. The secondary structure consensus of these sequences revealed that they all contain an N-terminal α1-helix with an average length of 51 aa (Fig. 1*C*), in agreement with a previous study (4). The amino acid consensus sequence revealed that the N-terminal portion of these α1-helices is highly conserved and predominantly hydrophobic, while the C-terminal portion is much less conserved (Fig. 1*C*). Comparison of these two regions to the α1 region of major pilins of solved T4aP structures shows that they correspond well to the hydrophobic α1-N and the amphipathic α1-C (Fig. 1*C*). The conservation of the Pro22 residue, emphasizes the importance of this residue in major T4a pilins (23). Thus, the size difference among major pilins arises from size differences in the globular domain.

### Cryo-EM Structure of the *M. xanthus* T4aP Reveals Unusual Tight Packing of PilA Subunits.

**Fig. 2.**
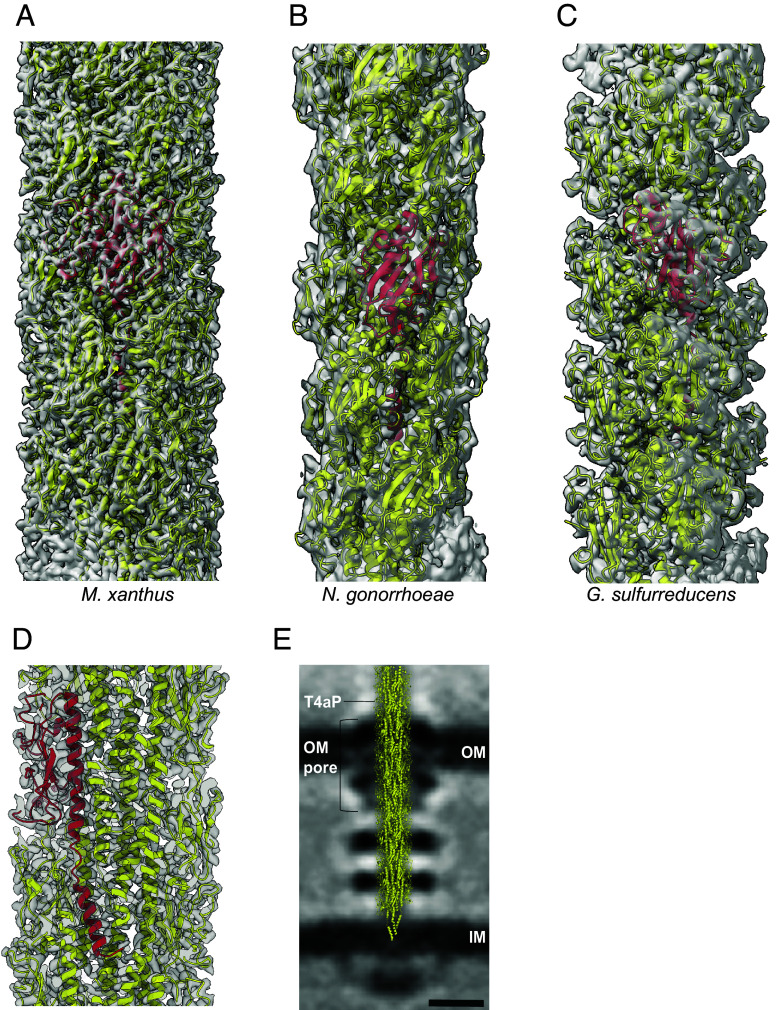
Cryo-EM structure of the T4aP^Mx^. (*A*–*C*) Comparison of cryo-EM reconstructions of T4aP and ribbon models of pilins from *M. xanthus* (*A*), *N. gonorrhoeae* (*B*), and the 2-chain G. *sulfurreducens* (*C*), where the transparent surfaces are the cryo-EM density maps, and one pilin subunit is shown in red in each. (*D*) Cross-section of the cryo-EM reconstruction of the T4aP^Mx^ with a single PilA subunit shown in red. (*E*) Placement of the T4aP^Mx^ structure into the central slice of the subtomogram average of the piliated *M. xanthus* T4aPM revealed by cryo-electron tomography (12) by superposition. The T4aP, the outer membrane pore (OM pore), OM, and IM are indicated. (Scale bar, 10 nm.)

To understand the properties of T4aP built from a large major pilin, we focused on T4aP^Mx^. The mature PilA (MXAN_5783) has a length of 208 aa with a predicted α1-N highly similar in sequence to the major pilins of the solved T4aP structures (Fig. 1*C*). For structure determination of T4aP^Mx^ (Fig. 2*A*), we purified T4aP from the hyperpiliated Δ*pilT* strain, in which T4aP are extended but not retracted, thereby allowing the purification of T4aP of sufficient purity and quantity for structural analyses (*SI Appendix*, Fig. S2 *A*–*C*). We used cryo-EM to determine the structure of the T4aP^Mx^ and obtained the structure at 3.0 Å resolution, the highest resolution so far reported for a T4aP structure (Fig. 2*A* and *SI Appendix*, Fig. S2*D*).

The subunits in the filament are related to each other by an azimuthal rotation of 100.7° and an axial rise per subunit of 10.0 Å, generating a right-handed 1-start helix with a pitch of ~36 Å and 3.6 subunits per turn, as determined from the power spectrum of the filaments (*SI Appendix*, Fig. S2*E*). The filaments are ~7 nm in diameter and, in contrast to all previous T4aP structures, resemble a rather solid cylinder without the corrugation of the surface observed in other T4aP structures (Fig. 2 *A*–*C* and *SI Appendix*, Fig. S3*A*). The individual PilA subunits within the cryo-EM reconstruction follow the overall blueprint of major pilins in solved T4aP structures with the N-terminal α1 generating the core of the pilus and the globular C-terminal domain decorating the surface (Fig. 2*D*). We also note that the solved structure of the T4aP^Mx^ with its diameter of ~7 nm readily fits into the overall architecture of the *M. xanthus* T4aPM, which we previously solved using cryo-electron tomography (12) (Fig. 2*E*).

**Fig. 3.**
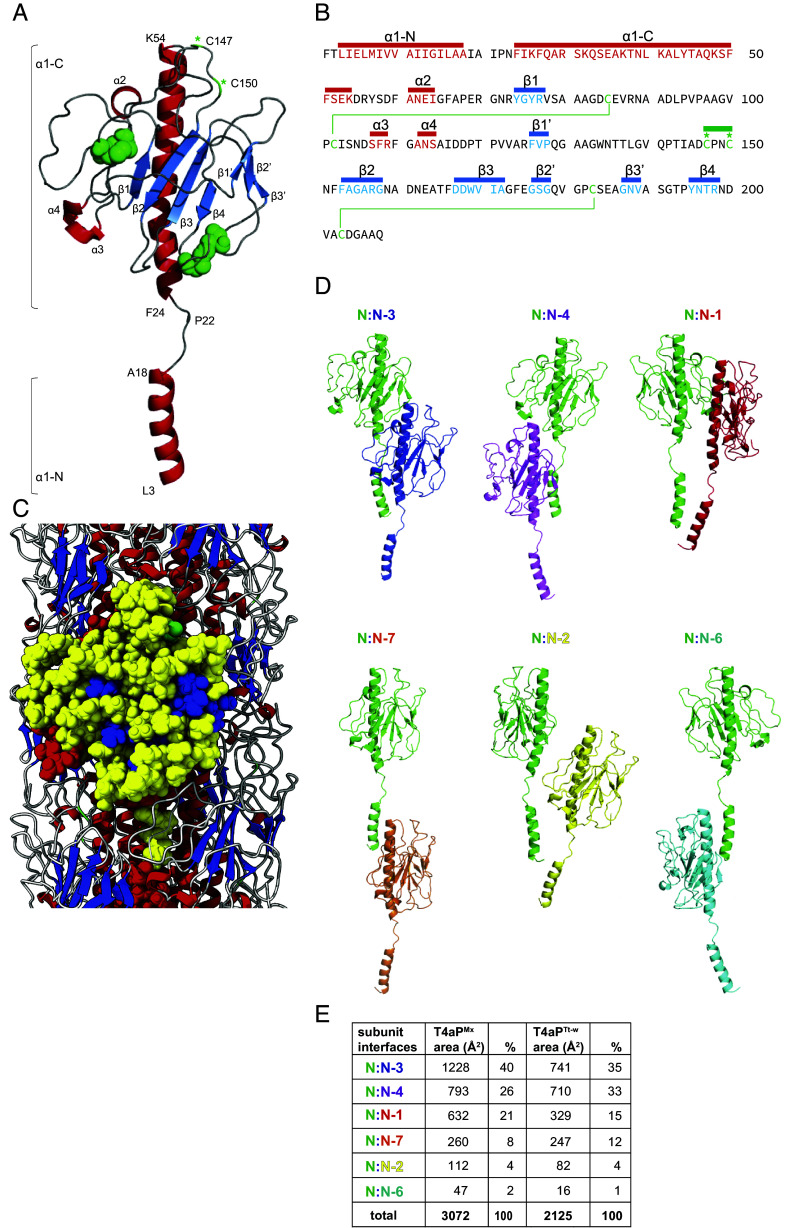
Atomic model of PilA^Mx^ and subunit interface analysis within the T4aP^Mx^ filament. (*A*) Ribbon representation of PilA^Mx^, with helical elements (α1-4) shown in red, β-stranded elements (β 1-4, β1′-3′) in blue, less-structured areas in gray, disulfide bridges in green, and free cysteines shown with green stars. The first and last residues of the helices of α1-N and α1-C as well as the conserved Pro 22 within the melted region are shown. (*B*) Sequence of PilA depicting structural elements, disulfide bridges, and free cysteines as in *A*. The green line indicates the CPxC motif, which is present in a subset of large pilins from Myxococcota (*SI Appendix*, Fig. S1). (*C*) Ribbon representation of the T4aP^Mx^ with structural elements shown in red, blue, and gray as in *A*. The surface of a single PilA subunit is shown in yellow (less-structured), red (α-helical), and blue (β-stranded) and with the surface exposed C150 residue in green. (*D*) Ribbon representation of the six different subunit interfaces with the largest three on *Top* and the three smaller ones at the *Bottom*. The color code of the subunits is shown above. (*E*) Areas of the six different subunit interfaces of T4aP^Mx^ in comparison to the corresponding interfaces in the wide T4aP of *T. thermophilus* and percentage of those different areas out of the total interface.

The 3.0 Å-resolution of the T4aP^Mx^ structure allowed for building de novo an atomic model of individual PilA subunits (Figs. 2*D* and 3*A*). The N-terminal α1-helix of PilA^Mx^ extends from residues 3 to 54 and contains the hydrophobic α1-N (aa 3 to 18) and the amphipathic α1-C (aa 24 to 54) separated by an unfolded stretch of five residues around the conserved Pro22 (Figs. 1*C*, 2*D*, and 3 *A* and *B* and *SI Appendix*, Fig. S3*B*). This is similar to the local melting of this helix seen in major pilins in solved T4aP structures (46) (Fig. 1*B* and *SI Appendix*, Fig. S3*B*).

The large globular domain (aa 55 to 208) contains two antiparallel β-sheets, one four-stranded sheet composed of β1-4 and one three-stranded sheet composed of β1′-3′, as well as three α-helices (α2-4) (Fig. 3 *A* and *B*). These regular structural elements (α-helices, β-strands), which are interrupted by loops, account for ~26% of the globular domain (*SI Appendix*, Fig. S3*B*). Compared to the major pilins of the other solved T4aP structures, the extra residues in PilA^Mx^ are largely found in a region between β1 and α3 and a region forming β3, β2′, and β3′ (*SI Appendix*, Fig. S3*C*), and while those previous structures all have a contiguous antiparallel three- (17) or four-stranded β-sheet (21, 23–26), the two antiparallel β-sheets in PilA^Mx^ are noncontiguous (Fig. 3 *A* and *B* and *SI Appendix*, Fig. S3 *B* and *C*).

Two disulfide bridges are present in the large globular domain. C95/C102 connects and likely stabilizes the region between β1 and α3. C183/C203 connects the β’-sheet to the C-terminal part of the globular domain (Fig. 3 *A* and *B*), somewhat similar to the C-terminal D-region known from other major T4a pilins that attaches the β-sheet to the C-terminal portion of the globular domain (4). Unlike the major pilins in other T4aP structures, PilA^Mx^ contains two Cys residues (C147 and C150) that do not engage in disulfide bridge formation (Fig. 3 *A* and *B* and *SI Appendix*, Fig. S3*C*). Of those two residues, only C150 is exposed on the surface (Fig. 3*C*). Labeling of T4aP^Mx^ with maleimide fluorescent dyes, which depends on a free thiol, were unsuccessful (47), therefore, we speculate that the surface-exposed C150 is posttranslationally modified so as to be nonreactive (48). Interestingly, ~9% of the large major pilins have six or more cysteine residues (20 out of 226) and 11 of these are from the Myxococcota (*SI Appendix*, Fig. S1 and Dataset S1). These myxobacterial pilins all contain a CPxC motif at a position similar to C147 and C150 of PilA^Mx^ (Fig. 3*B*).

### The Large Globular Domain Is Involved in Extensive Intersubunit Interactions.

Within the T4aP^Mx^ the globular domain of an individual PilA monomer extensively interacts with neighboring subunits (Fig. 3*D*), including six different subunit–subunit interfaces, three large (N:N-3, N:N-4, N:N-1) and three small (N:N-7, N:N-2, N:N-6) (Fig. 3 *D* and *E*). In total, these interfaces add up to ~3,000 Å^2^ of buried surface area (Fig. 3*E*). Because a pilin subunit interacts with pilins above and below, every individual pilin subunit has a total of 12 interaction partners, adding up to a total of ~6,000 Å^2^ of buried surface area per pilin.

For direct comparison of subunit interfaces, we used the structure of the wide T4aP of *T. thermophilus* (T4aP^Tt-w^), which is composed of the 125 aa PilA4 pilin and was previously solved at a high resolution (*SI Appendix*, Fig. S3*A*) (25). T4aP^Tt-w^ also has six subunit interfaces and similarly to the T4aP^Mx^, the largest interfaces occur between N:N-3, N:N-4, and N:N-1 (Fig. 3*E*). In comparison to the T4aP^Tt-w^, there is a ~50% increase in the buried interfacial area per subunit in the T4aP^Mx^ (Fig. 3*E*), deriving largely from more extensive interactions in the N:N-3 and N:N-1 interfaces. Similarly, a comparison of the N:N-3, N:N-4, and N:N-1 interfaces of T4aP^Mx^ with those of the lower resolution T4aP structures of the *E. coli* EHEC (major pilin, 140aa), *N. gonorrhoeae* (major pilin, 158aa), *N. meningitidis* (major pilin, 161aa), and *P. aeruginosa* PAK (major pilin, 143aa) shows that these three interfaces in these four structures vary from ~1,500 to 2,000 Å^2^ (21), and are thus also significantly smaller than in the T4aP^Mx^.

### A Structural Model of the Complete T4aP^Mx^ Including the Tip Complex.

T4aP are capped by tip complexes comprised of minor pilins and sometimes also the PilY1 adhesin (13, 15–19). Similar to major pilins, minor pilins are composed of an N-terminal α1-helix and a globular C-terminal domain (4); PilY1 proteins share a conserved C-terminal domain while the N-terminal domain is more variable (49).

Recently, a complete structural model of the T4aP of *S. sanguinis* was suggested based on the solved structure of the pilus filament and a computational model of the three minor pilins PilA, PilB, and PilC (17). In this model, a PilB monomer connects a heterodimeric complex of PilA/PilC to the pilus filament. In this trimeric complex, the large adhesion domains of PilB, which contains a vWA domain, and PilC, which contains a lectin domain, make up the ultimate pilus tip, allowing this complex to mediate binding to host cells (17).

In *M. xanthus*, the tip complex is composed of four minor pilins together with the PilY1 protein (13, 15, 16). To obtain a model of the complete T4aP^Mx^ with a tip complex composed of these five proteins, we generated structural models based on the solved T4aP^Mx^ structure and AlphaFold-Multimer models of complexes composed of the major pilin PilA, the four minor pilins and PilY1. *M. xanthus* encodes three sets of each four minor pilins and one PilY1 adhesin (13, 15, 16). For two of these three sets, i.e., those encoded by gene cluster_1 and gene cluster_3, experimental data support that they form a tip complex involved in adhesion (13, 15, 16). For the complex formed by the cluster_3 proteins, it was proposed that the less conserved N-terminal domain of PilY1.3, sits at the tip while the conserved C-terminal domain interacts with a complex composed of four minor pilins below, which, in turn, interacts with PilA below (13). Specifically, based on pull-down experiments and direct interaction analyses, the minor pilin PilX3 was placed directly below PilY1.3, followed by PilW3, FimU3, PilV3, and PilA (13).

**Fig. 4.**
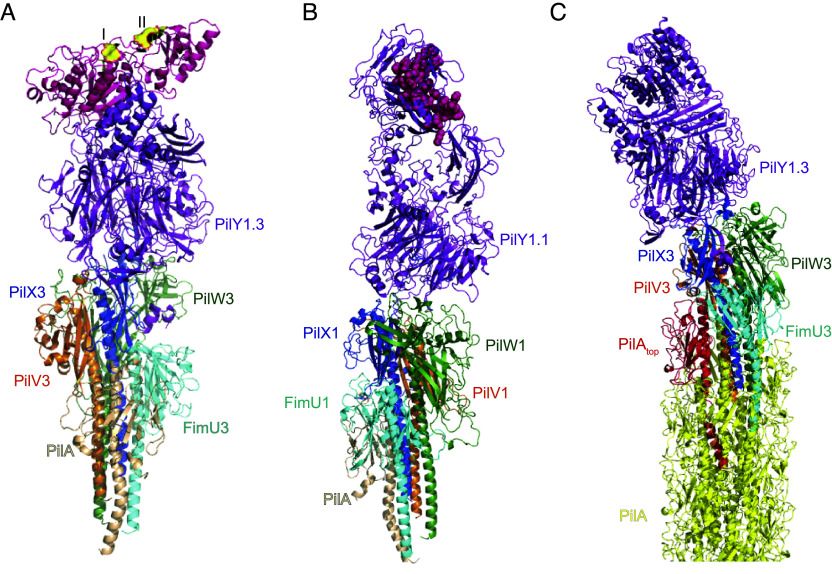
Complete T4aP^Mx^ structure composed of T4aP^Mx^ and a tip complex. (*A*) AlphaFold-Multimer model of a complex composed of PilY1.3, four minor pilins (FimU3, PilV3, PilW3, PilX3) and PilA in the indicated colors (model 1, pTM 0.79, ipTM 0.88, *SI Appendix*, Fig. S4*A*). The N-terminal vWA-domain (aa 35 to 436) is shown in pink and the two predicted MIDAS motifs (I: D_42_-x-S_44_-x-S_46_; II: D_112_-x-S_114_-x-S_116_) are shown as yellow spheres. Residue numbers refer to PilY1.3 without its signal peptide. (*B*) AlphaFold-Multimer model of a complex composed of PilY1.1, four minor pilins (FimU1, PilV1, PilW1, PilX1) and PilA in the indicated colors (model 1, pTM 0.67, ipTM 0.88, *SI Appendix*, Fig. S4*B*). The N-terminal DUF4414 domain (aa 458 to 506) is shown as pink spheres. Residue numbers refer to PilY1.1 without its signal peptide. (*C*) Complete T4aP^Mx^ structure composed of T4aP^Mx^ and a tip complex. The complete T4aP^Mx^ structure was generated by superposing the top PilA (red) of the T4aP^Mx^ (otherwise yellow, as in Fig. 2*E*) with the bottom PilA (wheat) of the AlphaFold-Multimer model as in *A* (RMSD = 0.841). The superposed PilA from the AlphaFold-Multimer model is not shown in the complete T4aP^Mx^ structure.

A high confidence AlphaFold-Multimer model of the cluster_3 proteins (model 1, pTM 0.79, ipTM 0.88) (Fig. 4*A* and *SI Appendix*, Fig. S4*A*) was consistent with the suggested organization of this complex with PilY1.3 at the tip followed by PilX3, PilV3, PilW3, FimU3, and PilA at the base, i.e., the only difference is the placement of PilV3 between PilW3 and PilX3 (Fig. 4*A*). Interestingly, despite substantial sequence diversity between cluster_3 and cluster_1 components (13, 16), the same order of components is predicted for the high confidence AlphaFold-Multimer model of the cluster_1 proteins (model 1, pTM 0.67, ipTM 0.88) (Fig. 4*B* and *SI Appendix*, Fig. S4*B*). In both AlphaFold-Multimer models, only α1 of PilA is slightly kinked (around the conserved P22), while the four minor pilins of each cluster lack that residue (13), and their α1-helices are relatively straight (Fig. 4 *A* and *B*). As also seen for the *S. sanguinis* T4aP tip complex model (17), AlphaFold does not predict melting of α1 for the major pilin (Fig. 4 *A* and *B*). In both models, the less conserved N-terminal domains of the PilY1 proteins, which contain either a putative lectin domain DUF 4114 (PilY1.1) (15), or a vWA-domain (PilY1.3) (13) make the ultimate tip (Fig. 4 *A* and *B*). The vWA-domain of PilY1.3 contains two MIDAS (metal ion-dependent adhesion site) motifs (Fig. 4*A*), which are reported to promote the adhesive properties of proteins upon coordination of a divalent metal ion together with an acidic residue of the ligand (50, 51).

Interestingly, a stretch of eight residues of the C terminus of PilY1.3 (aa 1444 to 1451, LQNVYELP) is modeled to form a β-strand, which, together with two β-strands of PilX3, forms a three-stranded antiparallel β-sheet (*SI Appendix*, Fig. S4*C*), suggesting that PilX3 and PilY1.3 interact by β-strand addition (52). A similar β-strand addition between PilX1 and PilY1.1 is also predicted for the model of the cluster_1 proteins (*SI Appendix*, Fig. S4*D*). In that case, a stretch of eight residues at the C terminus of PilY1.1 (aa 1379 to 1386, SKVLIYAP) is modeled to form a β-strand, which interacts with three β-strands of PilX1 forming a four-stranded antiparallel β-sheet (*SI Appendix*, Fig. S4*D*). Intriguingly, protein–protein interactions by β-strand addition are also involved in the assembly and stabilization of the Type 1 pilus (53) and are reported to be extraordinarily stable against dissociation and unfolding (54).

To generate the complete model of the T4aP^Mx^ including the tip complex, we fitted the model of the tip complex of the cluster_3 proteins into the T4aP^Mx^ structure by superposing the top PilA of the T4aP^Mx^ with the PilA of the AlphaFold-Multimer model (Fig. 4*C*). Importantly, these two PilA molecules could readily be superposed giving rise to a structure in which the four minor pilins caps the T4aP^Mx^ followed by the PilY1.3 adhesin at the tip and firmly attached through its C-terminal domain to PilX3 via β-strand addition.

### T4aP^Mx^ Has Increased Bending and Axial Stiffness Compared to Less Tightly Packed T4aP.

Since the resistance to bending will scale as the fourth power of the radial mass distribution, we expected that the tight packing and the increased contacts between the outer domains near the outside of the pilus would make the T4aP^Mx^ filament more rigid than previously studied ones. We quantified its bending stiffness with the persistence length. Because the persistence length (PL) is derived from an analysis of fluctuations in curvature from filaments at thermodynamic equilibrium, cryo-EM is ill-suited for making such measurements, due to the large forces present from both fluid flow during blotting and the compression of long filaments into a thin film (55, 56). In contrast, in the negative staining procedure the filaments are tightly adsorbed to a grid before blotting-induced shear forces occur, and it has been shown that adsorption of flexible filaments to a two-dimensional surface can capture an equilibrium distribution (57). Consequently, we used purified T4aP^Mx^ from WT and Δ*pilT* cells visualized by negative stain transmission electron microscopy (TEM) and determined the PL. As expected, T4aP^Mx^ from these two strains had the same PL (21 µm) (*SI Appendix*, Figs. S3*A* and S5*A*). In parallel experiments, we determined the PL of the less tightly packed T4aP of *N. gonorrhoeae* (T4aP^Ng^, major pilin, 158aa) and *P. aeruginosa* PAK (T4aP^Pa^, major pilin, 144aa) as 11 µm and 13 µm, respectively (*SI Appendix*, Figs. S3*A* and S5*A*). The size of the major pilins as well as the surface corrugation in the T4aP^Ng^ and T4aP^Pa^ differ (*SI Appendix*, Fig. S3*A*), and we suggest that together this explains the small difference in their PL (*SI Appendix*, Figs. S3*A* and S5*A*). In total, we suggest that the tight packing of the large major pilin in T4aP^Mx^ results in the increased bending stiffness.

**Fig. 5.**
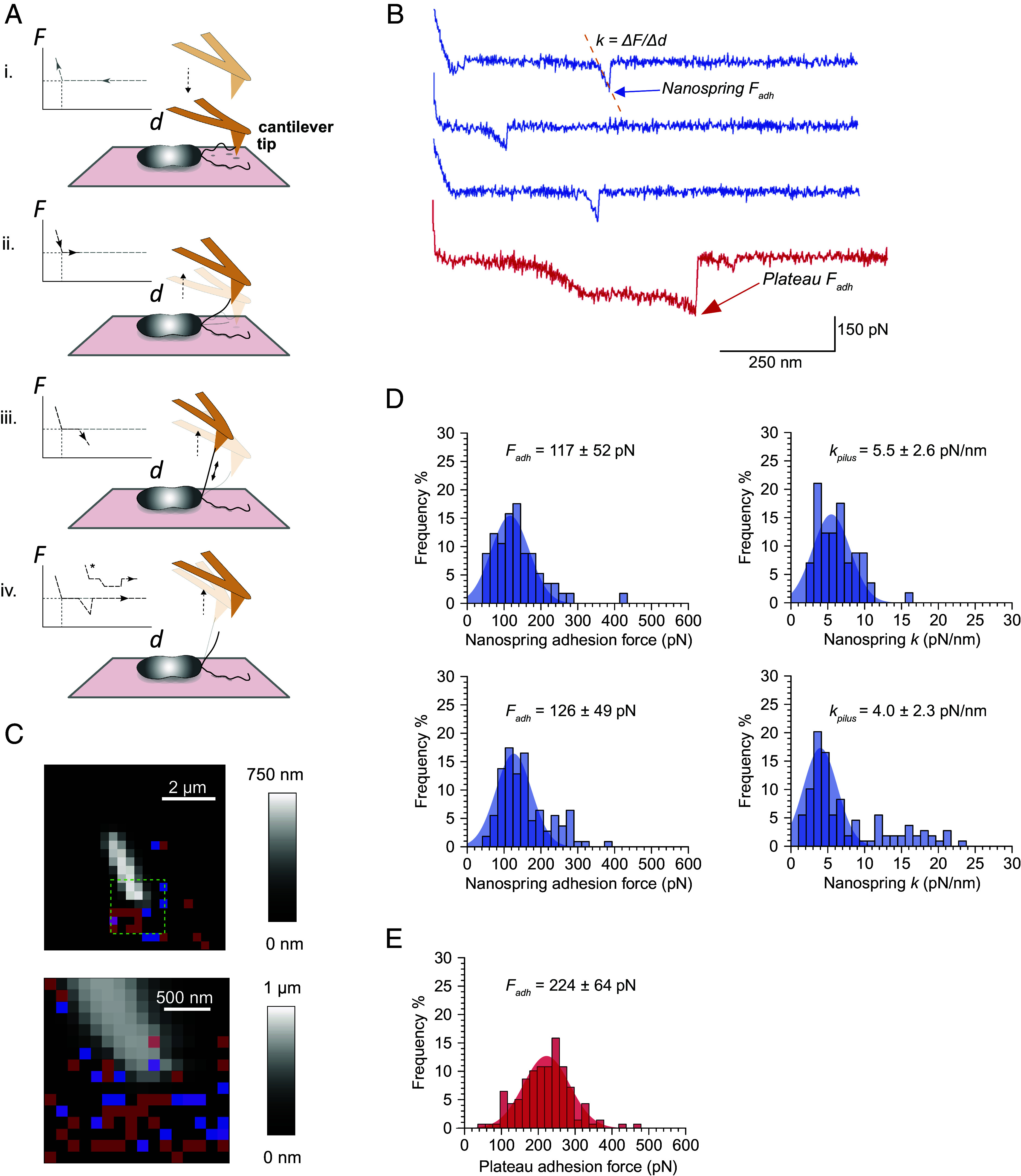
T4aP^Mx^ have increased axial stiffness and elastic properties and undergo force-induced elongation in response to pulling forces by AFM-FS. (*A*) Schematic of the AFM-FS approach to collect force-extension profiles of *M. xanthus* T4aP. (*i*) Cells in buffer were adhered to a polystyrene surface. The hydrophobic AFM probe on the cantilever approached toward the surface near one of the cell poles until it touches and pushes on the surface of the dish or cell, causing the upward bending of the cantilever that is recorded as a positive force in the approach Force-distance (*F*-*d*) curve (gray). By chance, a T4aP freely floating in the buffer adheres to the hydrophobic tip (i.e., hydrophobic contacts are established between the solvent accessible surface of the pilus fiber and the CH_3_ groups exposed on the AFM tip). (*ii*) The AFM tip is retracted away from the surface lifting the relaxed T4aP until (*iii*) the T4aP becomes loaded with tension resulting in downward bending of the cantilever, thereby allowing quantification of the tensile force (black *F*-*d* curve). (*iv*). When the tensile force within the T4aP exceeds that of the bonds between the pilus and the hydrophobic AFM tip, the contact between the two is ruptured and the cantilever relaxes resulting in a zero-force measurement (baseline) in the *F*-*d* curve. Two types of signatures are expected for T4aP, linear (Hookean) nanosprings and constant force plateaus (indicated with an asterisk in the *F*-*d* curve). (*B*) Three representative *F*-*d* curves obtained for WT *M. xanthus* cell showing nanospring signatures (blue). The rupture/adhesion force (*F_adh_*) is indicated by a blue arrow, the dotted line indicates the slope of the nanospring profile used to determine its spring constant (*k_pilus_*). Representative *F-d* curve obtained for a *M. xanthus* WT cell showing a force plateau signature (red). The rupture/adhesion force (*F_adh_)* is indicated by a red arrow. (*C*) *M. xanthus* WT cells probed in force volume mode in which an *F-d* curve is recorded at each pixel of a rectangular raster grid using a constant approach and retract velocity. Shown is a representative height map of a whole cell (*Top*) and zoom of the lower pole (*Bottom*, 16 × 16 pixels). The overlaid red, blue, and purple pixels indicate where force plateau, nanospring or both signatures were detected. (*D*) Histograms showing the distribution of *F*_adh_ (*Left*) and nanosprings *k_pilus_* (*Right*) as determined from *F*-*d* curves generated at a probe retraction velocity of 1 µm/s (*Top*, n = 57 nanosprings in 82/1312 curves from five tip-cell combinations) or 5 µm/s (*Bottom*, n = 109 nanosprings in 44/5,400 curves from five tip-cell combinations). Numbers indicate mean ± SD. (*E*) Histogram showing the distribution of plateau *F_adh_* as determined from *F*-*d* curves generated at a probe retraction velocity of 5 µm/s (n = 141 force plateaus in 125/5,400 curves from five tip-cell combinations). The number indicates mean ± SD.

We also expected that the increased subunit contact interfaces would increase the axial rigidity of T4aP^Mx^, resulting in a greater force needed to extend these filaments. We, therefore, analyzed the force-extension behavior and adhesive properties of T4aP^Mx^ in live cells using atomic force microscopy (AFM) force spectroscopy (FS) as described for T4aP in *P. aeruginosa* PAO1 and PA14 (major pilin length:143 & 173 aa, respectively) (31, 33, 58). Those studies reported two distinct force-extension profiles when single T4aP on live cells were pulled: i) a tensile force that initially increased in an approximately linear fashion with pilus stretching before rupturing of the contact between the pilus and the AFM tip; and ii) an initial increase in force followed by a constant force plateau before rupture occurred. Because of the approximate linearity of the first type, these profiles were called linear nanosprings, and their spring constant *k_pilus_*, which is a measure of pilus axial stiffness, was quantified as ~2 pN/nm (31, 33). We applied the AFM-FS methodology used in ref. 33 to characterize T4aP^Mx^ on live cells (Fig. 5*A* and *SI Appendix, Methods*). As previously observed for *P. aeruginosa* T4aP, we observed both nanospring (Fig. 5*B*, blue traces) and force plateau signatures (Fig. 5*B*, red trace) almost exclusively close to one of the cell poles for wild-type (WT) *M. xanthus* cells (Fig. 5*C*). By contrast, such signatures were nearly absent in *M. xanthus* Δ*pilA* cells (*SI Appendix*, Fig. S5*B*). The measured rupture forces in the pilus nanosprings (~120 pN) (Fig. 5*D*) and force plateaus (~220 pN) (Fig. 5*E*) were similar to those reported for *P. aeruginosa* T4aP (31, 33, 58), indicating no differences in the adhesive properties of *M. xanthus* and *P. aeruginosa* pili. The spring constant *k_pilus_* of T4aP^Mx^ at low (1 µm/s as in ref. 33) and at moderate (5 µm/s as in refs. 31 and 58) pulling speeds was ~5.5 pN/nm and ~4.0 pN/nm, respectively (Fig. 5*D*). Importantly, these values are at least twofold higher than those reported for T4aP in *P. aeruginosa* PAO1 and PA14 (31, 33), indicating a greater average axial stiffness of the T4aP^Mx^ and that T4aP^Mx^ are more resistant to stretching than T4aP of *P. aeruginosa*.

We also note that even though the mean rupture forces in the pilus nanosprings (Fig. 5*D*) and force plateaus (Fig. 5*E*) were in close agreement with the reported values for *P. aeruginosa* T4aP, T4aP^Mx^ can resist pulling forces up to 400 to 500 pN before the contact between the pilus and the AFM tip ruptures (Fig. 5*E*) while T4aP of *P. aeruginosa* resisted forces only up to 250 pN (33). Nevertheless, T4aP^Mx^ have elastic properties and undergo a force-induced elongation in response to pulling forces as previously described for T4aP of *N. gonorrhoeae, N. meningitidis,* and *P. aeruginosa* (31–34) consistent with the previous hypothesis that the extensibility arises from further melting of the N-terminal α-helix (23).

### Modifying the Interface between PilA Subunits by Mutagenesis Reduces Pilus Bending Stiffness, T4aP-Dependent Motility, and Pilus Length.

**Fig. 6.**
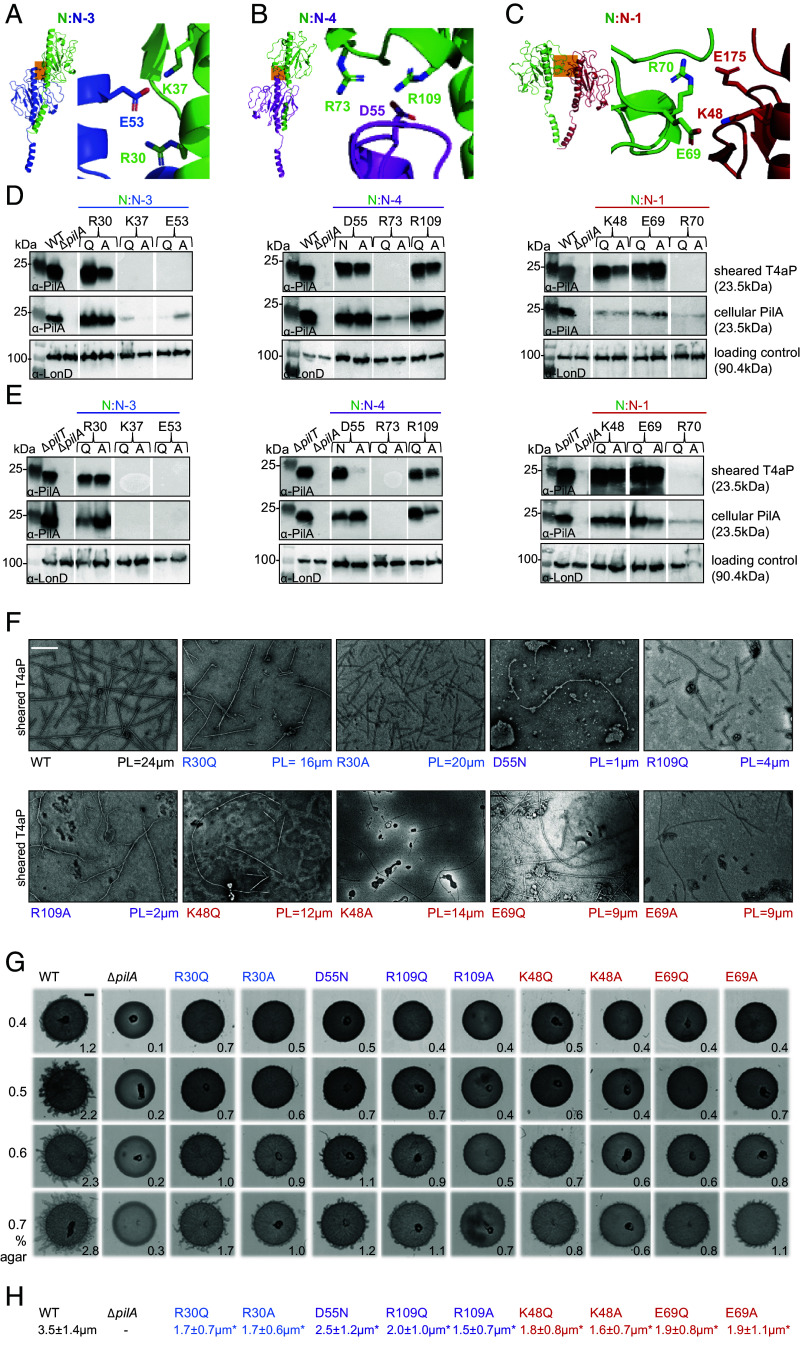
Mutagenesis of selected charged residues in PilA subunit interfaces. (*A*–*C*) Ribbon representation of interacting subunits N:N-3 (*A*), N:N-4 (*B*), and N:N-1 (*C*) as overviews (*Left* parts) with orange boxes indicating the position of the magnified views (*Right* parts) depicting the charged residues in these areas. The depicted residues, except for E175, were all targeted for mutagenesis. The subunit color code is as in Fig. 3*D*. (*D* and *E*) Effect of amino acid substitutions at intersubunit interfaces on PilA* accumulation and T4aP formation. T4aP sheared off from the surface of cells (*Top* rows), and total cell extracts (*Middle* rows) were separated by SDS-PAGE and probed with α-PilA antibodies. The membrane used for cell extracts was stripped, and probed with α-LonD antibodies as a loading control (*Bottom* rows). The PilA variants were analyzed in WT (*D*) and the Δ*pilT* mutant (*E*). T4aP sheared and purified from the same amount of cells of the parent strain (WT or Δ*pilT*) were loaded and separated by SDS-PAGE. For WT and its derivatives, T4aP sheared from ~15 mg cells, and cellular fractions from ~0.4 mg cells were loaded per lane. For the hyperpiliated Δ*pilT* and its derivatives, T4aP sheared from ~0.6 mg cells, and cell extracts from ~0.1 mg were loaded per lane. Proteins with their calculated molecular masses and positions of molecular markers are indicated. Gaps between lanes indicate lanes that were deleted for presentation purposes. (*F*) Substitutions at intersubunit interfaces reduce the persistence length of T4aP. Negative staining electron micrographs of sheared and precipitated T4aP. PL values in µm are shown below the images. (Scale bar, 250 nm.) Note that these pili, in contrast to those in *SI Appendix*, Fig. S2*C*, were imaged directly after the precipitation and not further purified by sucrose gradient centrifugation. (*G*) Substitutions at intersubunit interfaces perturb T4aP-dependent motility. Cells were incubated 24 h before imaging. The Δ*pilA* mutant served as a negative control. (Scale bar, 1 mm.) Numbers indicate increase in colony diameter (mm) in 24 h. The shown experiment is one of two independent experiments with similar results. (*H*) Substitutions at intersubunit interfaces reduce the length of T4aP in live cells. T4aP were imaged in the WT background by TEM and negative staining electron micrographs of cells from indicated strains analyzed. For each strain, the T4aP length from three cells was measured. The mean T4aP length ± SD is shown in µm. *, significant difference in comparison to WT T4aP using Student’s *t* test (*P*-value < 0.05).

Among the six interfaces between PilA subunits in the T4aP^Mx^, N:N-3, N:N-4, and N:N-1 are not only the largest contributors to the subunit interface but also significantly more extensive than those in the T4aP^Tt-w^ (Fig. 3 *D* and *E*), and the T4aP of *E. coli* EHEC, *N. gonorrhoeae*, *N. meningitidis,* and *P. aeruginosa* PAK. To assess how these three interfaces contribute to pilus bending stiffness and to T4aP function in vivo, we mutagenized charged residues engaged in salt bridge formation in these three interfaces (Fig. 6 *A*–*C*). Specifically, we targeted the residues R30, K37, E53 at the N:N-3 interface, D55, R73, R109 at the N:N-4 interface, and K48, E69, R70 at the N:N-1 interface and substituted these residues separately with residues with either a polar side chain (Asn or Gln) or Ala. These nine residues are either localized in α1-C (R30, K37, K48, E53) or in the globular domain (D55, E69, R70, R73, R109) (Fig. 3*B*), and are forming salt bridges connecting either α1-helices (R30, K37, E53 at the N:N-3 interface), globular domains (D55, R73, R109 at the N:N-4 interface, R70, E175 at the N:N-1 interface), or α1-helices and globular domains (K48, E69 at the N:N-1) of the corresponding subunits (Fig. 6 *A*–*C*). The corresponding 18 mutations were introduced into the *pilA* gene at the native locus of the WT and in the retraction-deficient Δ*pilT* mutant to distinguish between T4aP extension and hyperretraction defects (i.e., T4aP extension followed by immediate retraction), caused by these substitutions.

We first examined the accumulation of the PilA variants in total cell extracts and their ability to support T4aP formation. Substitutions of four of the nine residues (K37, E53 of α1-C and R70, R73 of the globular domain) (Fig. 6 *A*–*C*) caused strongly reduced or abolished PilA* accumulation in total cell extracts of both strain backgrounds (Fig. 6 *D* and *E*). Consistently, in a T4aP shear-off assay in which pili are sheared off the surface of cells, these PilA variants did not support T4aP formation in either strain background (Fig. 6 *D* and *E*). Thus, these residues are important for PilA stability and lack of T4aP formation is neither due to an extension defect nor a hyperretraction defect.

Mutagenesis of the remaining five residues allowed PilA* accumulation in total cell extracts at the same or lower level compared to the level of PilA^WT^ in WT (Fig. 6*D*) and the Δ*pilT* mutant (Fig. 6*E*). Importantly, except for the D55A variant, they supported T4aP formation in both strain backgrounds at essentially the same level as PilA^WT^ (Fig. 6 *D* and *E*). Paradoxically, the D55A variant, while accumulating in both strain backgrounds, only supported T4aP formation in the WT but not in the Δ*pilT* mutant. From here on, we focused on the nine variants that accumulated and supported T4aP formation in both strain backgrounds.

The PL of T4aP^Mx^ purified from WT and the Δ*pilT* mutant have the same PL (*SI Appendix*, Fig. S5*A*). Therefore, to assess the mechanical properties of the nine variant T4aP, we purified them from the Δ*pilT* background and determined their PL (Fig. 6*F*). All nine variants had a moderately to strongly reduced PL (Fig. 6*F* and *SI Appendix*, Fig. S6*A*). In particular, substitutions in the N:N-4 interface caused dramatic reductions in PL (Fig. 6*F*), while substitutions in the N:N-3 and N:N-1 interfaces generally only caused a ~50% reduction in PL (Fig. 6*F*). We conclude that the substitutions do not interfere with the extension of T4aP; however, the T4aP assembled by the PilA variants have decreased bending stiffness.

To analyze whether PilA subunit interface alteration affects T4aP-dependent motility, we analyzed the *M. xanthus pilT*^+^ strains synthesizing these nine variants. *M. xanthus* moves by T4aP-dependent motility, which is favored on soft, moist surfaces, and by gliding motility, which is favored on hard agar (39). Surface stiffness was reported to stimulate T4aP-dependent motility in *P. aeruginosa* (59). Therefore, we tested WT as well as the strains expressing the nine PilA variants on soft agar of different stiffness by using a range of agar concentrations (0.4 to 0.7%) and the increase in colony diameter at 24 h as a readout for T4aP-dependent motility. The Δ*pilA* strain, which only moves by gliding motility, served as a negative control for T4aP-dependent motility and to verify that gliding motility did not significantly contribute to the increase in colony diameter under these conditions.

The WT displayed T4aP-dependent motility on all four agar surfaces generating the characteristic flares at the colony edge, and the colony diameter increased ~2.5-fold with the agar concentration, while the Δ*pilA* mutant, as expected, generated smooth-edged colonies and only displayed a minor increase in colony diameter (Fig. 6*G*). These findings are consistent with the observation that surface stiffness stimulates T4aP-dependent motility in *P. aeruginosa*. All strains expressing a PilA variant had strongly reduced T4aP-dependent motility at all agar concentrations (Fig. 6*G*). Like the WT, they generally showed improved T4aP-dependent motility with increasing agar concentrations; however, none reached the WT level even at 0.7% agar (Fig. 6*G*). Because the reduction in T4aP-dependent motility did not correlate with the reduction in PL (Fig. 6 *F* and *G*), we measured the absolute length of the pili made by PilA^WT^ and the PilA variants. The nine PilA variants made pili that were slightly but significantly shorter than those made by PilA^WT^ (Fig. 6*H*), however, we did not observe a correlation between the reduction in T4aP-dependent motility and pilus length (Fig. 6 *G* and *H*).

We conclude that WT T4aP^Mx^ supports T4aP-dependent motility on surfaces of different stiffnesses and more efficiently at higher agar concentrations. In contrast, the T4aP of the variants are shorter and less efficient at supporting motility under all the tested conditions and are only slightly stimulated on stiffer surfaces.

## Discussion

Here, we elucidate the structure of the T4aP^Mx^ using cryo-EM at a resolution of 3.0 Å and demonstrate that, in contrast with all previous T4aP structures, the T4aP^Mx^ structure is a tightly packed dense structure. The PL of T4aP^Mx^ is ~twofold higher than those of *P. aeruginosa* and *N. gonorrhoeae*, consistent with the greatly increased contacts at higher radius in the T4aP^Mx^. Similarly, the spring constants of T4aP^Mx^ at low and moderate pulling speeds are at least twofold higher than those reported for *P. aeruginosa* T4aP, indicating a greater axial stiffness of T4aP^Mx^. Also, T4aP^Mx^ can resist pulling forces up to 400 to 500 pN, in agreement with the observation that T4aP^Mx^ can resist forces up to 150 pN generated during retraction (9). These data make the T4aP^Mx^ the strongest and most rigid T4aP yet described. Nevertheless, T4aP^Mx^ have elastic properties and undergo a force-induced elongation in response to pulling forces as previously described for T4aP of *N. gonorrhoeae, N. meningitidis,* and *P. aeruginosa* (31–34).

Our structure suggests that the T4aP^Mx^ is more dense, more rigid, and stronger than other T4aP due to the tight-packing of the larger globular domains, which are involved in more extensive intermolecular interactions than seen in other T4aP structures. The larger C-terminal globular domains provide surfaces for extensive interactions, causing a measurable increase in total interface area, and allow every individual pilin to interact extensively with six pilins above and six below. Among these six interfaces, the three largest are the N:N-3, N:N-4, and N:N-1, and all three interfaces contain residues that engage in intersubunit salt bridges, i.e., R30_N_ and/or K37_N_ and E53_N-3_ in N:N-3, R73_N_ and/or R109_N_ and D55_N-4_ in N:N-4, and E69_N_ and K48_N-1_, R70_N_ and E175_N-1_ in N:N-1 (Fig. 5 *A*–*C*). Alteration of these three subunit interfaces reduced the bending stiffness of the corresponding T4aP^Mx^ variants but the disruption of the salt bridge connecting two globular domains in the N:N-4 interface had the strongest effect, supporting the hypothesis that the large globular domains contribute significantly to the increased bending stiffness.

We also observed that the PilA variants K37Q/A, E53Q/A, R70Q/A, and R73Q/A had reduced stability (Fig. 6 *D* and *E*), suggesting that these residues are important for intramolecular interactions before the incorporation of PilA into the T4aP and that mutagenesis of these residues causes misfolding and degradation of PilA*. This is similar to earlier findings, showing that mutagenesis of A18, I19 and A20 of PilA^Mx^ (I19 and A20 are part of the melted region, Fig. 3 *A* and *B*) can strongly affect PilA accumulation (60, 61). Interestingly, mutagenesis of other major T4a pilins also supports the notion that intra- and intersubunit salt bridges contribute to the stability of the pilin, assembly of the T4aP, and T4aP function (21, 23, 62).

We found that T4aP-dependent motility of *M. xanthus* was stimulated by increased substrate stiffness. A similar observation was made in *P. aeruginosa,* and it was suggested that this stimulation involves an increased probability of T4aP retraction on the stiffer agar surface (59). Interestingly, the PilA variants in which subunit interfaces were disrupted generally supported T4aP extension. However, the T4aP made from these PilA variants were less efficient at supporting T4aP-dependent motility than PilA^WT^ at all substrate stiffnesses. The T4aP made from the PilA variants had a reduced PL, indicating decreased bending stiffness or flexural rigidity. Similarly, the absolute length of these pili was slightly but significantly shorter than those made by PilA^WT^. However, neither the PL nor absolute pilus length correlated with the ability to support motility suggesting that it is neither the decreased bending stiffness nor the reduced pilus length per se that result in the motility defect. Accordingly, even variant T4aP with PLs similar to those of T4aP of *P. aeruginosa* and *N. gonorrhoeae* did not support motility. During the extension/adhesion/retraction cycles, only retraction generates a force sufficient to pull a cell forward (7, 8), suggesting that the variant T4aP likely have retraction defects. The hexameric PilB and PilT ATPases that power extension and retraction, respectively, bind at the base of the T4aPM in a mutually exclusive manner (12). The swap from PilB to PilT, and thus initiation of retraction, was suggested to be a stochastic event (63), or, alternatively, it was suggested that it is induced by adhesion of the pilus tip to the substratum in a process in which tip adhesion causes conformational changes in the pilus that are communicated to the base of the T4aPM (64). *N. gonorrhoeae* and *P. aeruginosa* T4aP, as also reported here for T4aP^Mx^, undergo force-induced conformational changes to elongate (31–33). Therefore, we speculate that the motility defect of the T4aP variants could be caused by 1) less efficient transmission of conformational changes from the tip to the base of the T4aPM to stimulate the swap from PilB to PilT, 2) reduced ability to undergo force-induced conformational changes during retraction, or 3) even breakage of the T4aP when it is pulled taut during retraction.

The T4aP^Mx^ structure is more dense due to the tight packing of the larger globular domains, resembling a solid cylinder without corrugation of the surface. Interestingly, the recent high-resolution structure of the *Vibrio cholerae* toxin coregulated T4b pilus (T4bP^Vc^), which is composed of the major pilin TcpA with a size of 192 aa, demonstrated that this pilus has a corrugated surface and is quite flexible (65). This comparison emphasizes that it is both the tight packing of the large major pilin and the large major pilin size that provide the distinct mechanical properties of the T4aP^Mx^. In future studies, it will be interesting to obtain structures of T4aP formed by large major pilins from different bacteria to further reveal the potential role of these distinct mechanical properties in T4aP-dependent function.

Our results advance our understanding of how sequence divergence of major T4a pilins shapes the functional properties of T4aP. Moreover, the information gained from the first complete T4aP model, composed of major pilins, four minor pilins, and a large PilY1 adhesin, provides insights into the interactions between major and minor pilins, as well as between minor pilins and PilY1, and might contribute to a better understanding of the distinct roles performed by these five proteins within the tip complex. In future studies of T4aP, it will also be interesting to obtain detailed insights into the interactions between the tip complex and the remaining T4aP.

## Materials and Methods

### Bacterial Strains, Plasmids, and Growth Media.

Strains and plasmids are listed in *SI Appendix*, Tables S3 and S4, respectively. All *M. xanthus* strains are derivatives of the DK1622 WT strain (66). In-frame deletion mutants were generated as described (67). All plasmids were verified by sequencing. All strains were confirmed by PCR and sequencing. Oligonucleotides are listed in *SI Appendix*, Table S5. *M. xanthus* strains were grown at 32 °C in 1% casitone broth (CTT) (1% casitone, 10 mM Tris-HCl pH 8.0, 1 mM KPO_4_ pH 7.6, and 8 mM MgSO_4_) or on 1% CTT, 1.5% agar plates supplemented with kanamycin (40 µg mL^−1^) when required (68). Growth was followed by measuring optical density at 550 nm (OD_550_). *E. coli* strains were grown in lysogeny broth (LB) (69). Plasmids were propagated in *E. coli* Mach1.

### Cryo-EM Sample Preparation and Data Collection.

Two μL of a T4aP^Mx^ sample was applied to plasma-cleaned lacey carbon grids, followed by plunge-freezing in liquid ethane using a Leica EM GP. Data collection was carried out at liquid nitrogen temperature on a Titan Krios microscope (Thermo Fisher Scientific) operated at an accelerating voltage of 300 kV. Using a K3 camera (Gatan), 40 movie frames were collected with a total dose of ~55 electron/Å^2^ and sampling of 1.08 Å/pixel. The movies were collected with defocus values ranging between −1 to −2.5 μm.

### Cryo-EM Image Processing and Reconstruction.

All the subsequent data processing was performed in CryoSPARC. Raw movie frames were used in the motion correction, followed by CTF estimation. Images with poor CTF estimation were eliminated. Filament tracer was used for filament picking and a total of 6,155,222 256 px-long segments were extracted from 13,006 CTF-corrected images. After final round of 2D classification, 1,312,119 segments remained and were subjected to homogenous refinement, which yield a map of recognizable secondary structure features when imposing the 1-start helical symmetry. The helical parameters converged to a twist of 100.7° and a rise of 10 Å per subunit. The resolution of the final reconstruction was determined by two independent half maps showing a resolution of 3.0 Å at FSC = 0.143.

### Model Building and Refinement.

An initial homologous model generated via SWISS-MODEL was docked into the cryo-EM map by rigid body fitting in Chimera (70) and manually edited the model in Coot (71). The modified monomeric model was then real-space refined using Phenix (72) to improve the stereochemistry as well as the model-map correlation coefficient. The refined monomeric model was rebuilt by RosettaCM with helical symmetry, followed by another three rounds of real-space refinement to reduce subunit clashes. The refined symmetrical model was validated with MolProbity (73), and the coordinates were deposited to the Protein Data Bank with the accession code 8TJ2. The corresponding cryo-EM map was deposited in the EMDB with accession code EMD-41298.

### Determination of Subunit Interface Areas.

The interfacial areas were determined by PDBePISA (https://www.ebi.ac.uk/pdbe/pisa/) (74).

Additional Material and Methods information regarding Bioinformatics, Dataset S1, AlphaFold-Multimer model building, Dataset S2, AFM tip functionalization and FS, T4aP purification and T4aP shearing assays, T4aP-dependent motility assays, antibodies and immunoblot analysis, transmission electron microscopy, persistence length, and T4aP length determination can be found in *SI Appendix*, *Materials and Methods*.

## Supplementary Material

Appendix 01 (PDF)

Dataset S01 (XLSX)

Dataset S02 (XLSX)

## Data Availability

All study data are included in the article and/or supporting information.
