# Supplementary material for: Tight-packing of large pilin subunits provides distinct structural and mechanical properties for the Myxococcus xanthus type IVa pilus
Source: Proc Natl Acad Sci U S A. 2024 Apr 16;121(17):e2321989121. doi: 10.1073/pnas.2321989121 (PMC11046646; doi:10.1073/pnas.2321989121)
Supplement: Supplementary file 1 — Appendix 01 (PDF) [file pnas.2321989121.sapp.pdf]

# Supporting Information for

# Tight-packing of large pilin subunits provides distinct structural and mechanical properties for the *Myxococcus xanthus* type IVa pilus

Anke Treuner-Lange[1,*], Weili Zheng[2,#], Albertus Viljoen[3], Steffi Lindow[1], Marco Herfurth[1], Yves F. Dufrêne[3], Lotte Søgaard-Andersen[1] and Edward H. Egelman[2,*]

[1] Department of Ecophysiology, Max Planck Institute for Terrestrial Microbiology, 35043 Marburg, Germany

[2] Department of Biochemistry and Molecular Genetics, University of Virginia School of Medicine, Charlottesville, VA 22903, USA

[3] Louvain Institute of Biomolecular Science and Technology, UCLouvain, B-1348 Louvain-la-Neuve, Belgium

[#] Present address: Nanoimaging Services, San Diego, CA 92121, USA

* Corresponding authors: anke.treunerlange@mpi-marburg.mpg.de, egelman@virginia.edu

**This file contains:**

Supplementary Materials and Methods
Supplementary Figures 1-6
Supplementary Tables 1-5
Supplementary References

**Supplementary Materials and Methods**

**Bioinformatics.** Sequences of the K02650 (type IV pilus assembly protein PilA) were extracted from the KEGG SSDB database (1). To filter out highly homologous sequences we used the cdhit program with a threshold of 90% sequence identity (2). The 2308 obtained sequences were analyzed for presence of a class 3 signal peptide (SPIII) and subsequently processed into the mature pilin form using SignalP (6.0) (3). The taxonomic classification of the remaining 2071 pilin sequences was collected from KEGG SSDB database and sequences without a bacterial classification as well as sequences from bacterial phyla, only represented by one genome, were excluded from the analysis. The 1955 remaining pilin sequences (Dataset S1) were analyzed with the PROMALS3D multiple sequence and structure alignment server (4) to obtain aa and secondary structure consensus sequences. Alignments were generated using T-Coffee (5) and the ClustalW output format (6). They were shaded using the BoxShade Server. Residues on black are conserved in ≥60% of the proteins, and residues on gray are similar in ≥60% of the proteins. For the phylogenetic tree, the ANCESCON tool (7) of the MPI bioinformatics Toolkit (8) was used. The phylogenetic tree was annotated using iTol (v6) (9). The MIDAS motifs (DxSxS) were identified manually. The domains of PilY1 proteins were identified using Smart (10).

**AlphaFold-Multimer model building.** Structures were predicted using AlphaFold-Multimer modeling via ColabFold (Version 1.5.0) (11-13). ColabFold was run locally using *colabfold_batch* with default settings on an Nvidia A100 graphics card with 40 GB memory. Five models were generated and ranked according to their pTM and iPTM scores (11). The predicted Local Distance Difference Test (pLDDT) and predicted Alignment Error (pAE) graphs of the five models generated by ColabFold were visualized as vector-graphics using a custom Matlab R2020a (The MathWorks) script (14). Per residue model accuracy was estimated based on pLDDT values (>90, high accuracy; 70-90, generally good accuracy; 50-70, low accuracy; <50, should not be interpreted) (12). Relative domain positions were validated by pAE. The pAE graphs indicate the expected position error at residue X if the predicted and true structures were aligned on residue Y; the lower the pAE value, the higher the accuracy of the relative position of residue pairs and, consequently, the relative position of domains/subunits/proteins (12). PyMOL version 2.4.1 (Schrödinger LLC) was used to analyze and visualize the models. The PDB-text files of the AlphaFold-Multimer models of the two tip complexes (models 1, Fig. 4 A, B) are shown in Dataset S2. Structure superposition of the top PilA of the $T4aP^{Mx}$ with the bottom PilA of the AlphaFold-Multimer model was done using the align function in PyMol (root mean square deviation=0.841). The protein sequences of the mature minor pilins and PilY1 proteins without their signal peptides as reported earlier (15), were used for generating the models.

**AFM tip functionalization and FS.** Gold AFM probes (PNP-TR, NanoWorld) were incubated overnight in a 1mM ethanolic 1-dodecanethiol solution to modify them with methyl groups and render them hydrophobic, then rinsed with ethanol and sterile water and kept in milliQ water at 4°C until use (no longer than 48 hrs). Early exponential-phase *M. xanthus* cultures ($OD_{550}$ ~ 0.5) grown in CTT at 32°C in the dark, were diluted in MC7 buffer (10 mM MOPS pH 7.6, 1 mM $CaCl_2$) and passaged gently through a 26 gauge needle to dissolve cell aggregates before seeding cells in a 35mm untreated polystyrene Petri dish (Corning). After 30 min incubation to allow cells to adhere, they were adequately adhering for high-quality AFM-FS, gently rinsed with MC7 buffer, and immediately used for AFM. Prior to any AFM measurements, the spring constant of the probe's cantilever was determined as reported previously (16) allowing for the accurate correlation between measured cantilever deflection and tensile force in stretched T4aP. AFM recordings were done at room temperature using a NanoWizard® 4 NanoScience AFM (JPK Instruments) in force mapping (volume) mode, which allows the recording of *F-d* curves in a pixel-by-pixel fashion over a defined surface area in a raster array. Single adherent *M. xanthus* cells were visualized with an inverted microscope and force probed in MC7 buffer with the hydrophobic AFM tip. For each tip-cell pair, a large (10×10 µm, 32 × 32 pixels) force map was first recorded to generate a topographical image of a whole cell and to find pilus signatures localized to one of the cell poles (17). Subsequently, a smaller (3×3 µm, 32×32 or 16×32 pixels) map was recorded over a piliated pole. Sample height could be determined from the approach section of each *F-d* curve, while the retract portion provided pilus forced extension and adhesive information. *F-d* curve analysis was performed using the JPK data processing software. The nanospring constant (given as $k_{pilus}$ below) was calculated using the serial spring equation: $\frac{1}{k_{eff}} = \frac{1}{k_{pilus}} + \frac{1}{k_s}$, with $k_{eff}$ (effective spring constant) equal to the slope of the linear region of a nanospring extension profile in an *F-d* curve and $k_s$ (spring constant of the cantilever sensor). Graphs pertaining to AFM data was generated using R Studio.

**T4aP purification and T4aP shearing assays.** For structural analysis and persistence length measurements, T4aP were sheared off from WT and the hyper-piliated Δ*pilT* strain using a modified protocol (15) based on the procedure of (18). Briefly, cells grown on 1% CTT, 1.5% agar plates (10 plates, 12x12 cm) for 2-3 days were gently scraped off the agar and resuspended in 4 ml/plate pili resuspension buffer (100mM Tris-HCl pH 7.6, 150mM NaCl). The pooled suspension was vortexed for 10 min at the highest speed and centrifuged for 20 min at 13,000 *g* at 4°C to remove cell debris. The supernatant was centrifuged twice for 10 min at 13,000 *g* at 4°C. T4aP in the cell-free supernatant were precipitated by adding 10× pili

precipitation buffer (final concentrations: 100mM $MgCl_2$, 500mM NaCl, 2% PEG 6000) for at least 3 hrs at 4°C. The solution was centrifuged for 30 min at 13,000 *g* at 4°C, and the pellet resuspended 1ml pili resuspension buffer. The pili solution was loaded on top of a centrifuge tube containing a 10-70% sucrose gradient (29 ml) of pili resuspension buffer. After 15 hrs centrifugation at 115,000xg in a swing bucket rotor (SW72Ti) at 4°C, the tube was punched at the bottom, and 1.5 ml fractions harvested and analyzed by SDS-PAGE using SDS-lysis buffer (10% (v/v) glycerol, 50mM Tris-HCl pH 6.8, 2mM EDTA, 2% (w/v) SDS, 100mM DTT, 0.01% bromphenol blue). To remove the sucrose, PilA-containing fractions were diluted 13.5 fold in pili resuspension buffer, and the solutions precipitated again with pili precipitation buffer (s.a.). The pili were resuspended in pili resuspension buffer. Only T4aP purified from the hyper-piliated Δ*pilT* strain were of sufficient purity and quantity for structural analyses.

For T4aP shearing assays, 60 mg cells grown on 1% CTT, 1.5% agar plates for 2-3 days were gently scraped off the agar and resuspended in pili resuspension buffer. Cell suspensions were vortexed for 10 min at the highest speed. Cells from a 100 µl aliquot were harvested, the pellet solved in 200 µl SDS lysis buffer, and immediately denatured at 95°C for 5 min. This represents the cellular fraction. The remaining suspension was centrifuged for 20 min at 13,000 *g* at 4°C. The supernatant was removed and centrifuged twice for 10 min at 13,000 *g* at 4°C to remove cell debris. T4aP in the cell-free supernatant was precipitated by adding 10× pili precipitation buffer for at least 3 hrs at 4°C. The solution was centrifuged for 30 min at 13,000 *g* at 4°C, and the pellet was resuspended in SDS lysis buffer (for immunoblotting) or in pili resuspension buffer for negative staining (1µl per mg vortexed cells). T4aP sheared and purified from the same amount of cells of the respective parent strain (WT or Δ*pilT*) were loaded and separated by SDS-PAGE. For WT and its derivatives T4aP sheared from ~15 mg cells and 40 µg of protein from cellular fractions (~0.4 mg cells) were loaded per lane. For the hyperpiliated Δ*pilT* and its derivatives T4aP sheared from ~0.6 mg cells and 10 µg of protein from cellular fractions (~0.1 mg) were loaded per lane.

**T4aP-dependent motility assays.** Cells from exponentially growing *M. xanthus* cultures were harvested and resuspended in 1% CTT to a calculated density of $7\times10^9$ cells $ml^{-1}$. 5µl of cell suspension were spotted on soft agar CTT plates (0.5% casitone, 10mM Tris-HCl pH 8.0, 1mM $KPO_4$ pH 7.6, 8mM $MgSO_4$, with the indicated concentrations of select agar (Invitrogen)) and incubated at 32°C for 24 hrs. Colony edges were imaged using a Leica MZ75 stereomicroscope with a Leica MC120 HD camera. Colony diameters are measured at time point zero and after 24

h up to the longest flares. Numbers in mm represents the increase in colony diameter within 24 hrs.

**Antibodies and immunoblot analysis.** Immunoblotting was done with rabbit, polyclonal α-PilA and α-LonD antibodies (15). As secondary antibodies goat, anti-rabbit immunoglobulin G peroxidase conjugate (Sigma-Aldrich, A8275) was used. Blots were developed using Luminata™ Western HRP substrate (Millipore).

**Transmission electron microscopy.** For negative-staining of pili ~5 µl of sheared T4aP solutions were applied on 300 mesh Formvar/carbon copper-grids. After 10 min, grids were washed twice with water and negative-staining was done with a solution based on an Organotungsten compound (Nano-W, Nanoprobes). For negative-staining of cells 300 mesh Formvar/carbon copper-grids were pretreated for 1 hr with each 50 µl of a freshly prepared chitosan dilution (100-fold dilution in water of chitosan stock solution=15mg/ml chitosan in 2M acetic acid). After 1 hr the chitosan solution was blotted away with filter paper and the grids were dried. An aliquot of exponentially growing *M. xanthus* cultures (5-20 µl) were put on top of a pretreated grid and cells were allowed to attach to the grid at 32°C in a humid chamber. After 3 hrs, grids were washed twice with water and negative-staining was done with a solution based on an Organotungsten compound (Nano-W, Nanoprobes). Grids were inspected with a JEM-1400 electron microscope (JEOL) at 100 kV.

**Persistence length determination.** To determine the flexibility of different T4aP, persistence length measurements were performed using micrographs of negatively stained T4aP from the indicated strains. For each strain, 30-50 filaments were traced using the ImageJ analysis tool (19). Persistence length (L) is determined by the statistical relationship of cos (θ) and contour length (λ), according to $\exp(-\lambda/L)=\langle\cos(\theta)\rangle$.

**Pilus length determination.** To determine the length of T4aP from WT and PilA-variants, micrographs of negatively stained cells from indicated strains were analyzed using the MetaMorph application (Meta Imaging Series 7.8). For each strain, length of T4aP from three cells were measured. The mean T4aP length ± standard deviation (STDEV) is shown in µm. Student’s t-test was used to test whether the mean T4aP length of the variants is significantly different from WT mean T4aP length ($p$-value $< 0.05$).

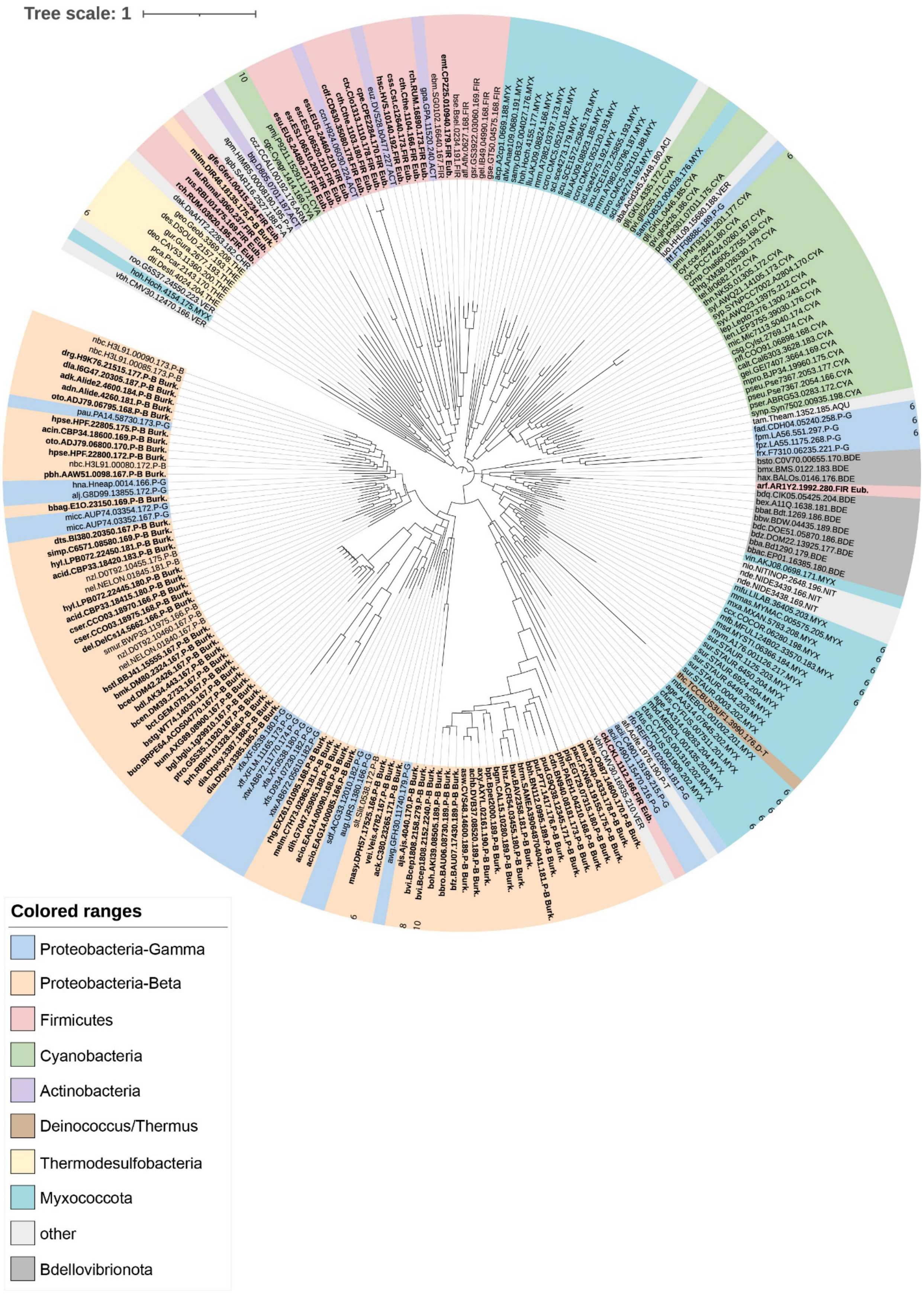
Tree scale: 1
Colored ranges
Proteobacteria-Gamma
Proteobacteria-Beta
Firmicutes
Cyanobacteria
Actinobacteria
Deinococcus/Thermus
Thermodesulfobacteria
Myxococcota
other
Bdellovibrionota

**Supplementary Fig. 1. Phylogenetic tree of 226 large major T4a pilins.**

Phylogenetic tree of 226 large major pilins, as listed in SI Appendix, Table S1. The large pilins of the Betaproteobacteria are found especially in Burkholderiales, and those are shown in bold with the abbreviation Burk. at the end of the locus tag. The numbers at the outer edge of the circle indicate large pilins with six or more cysteine residues.

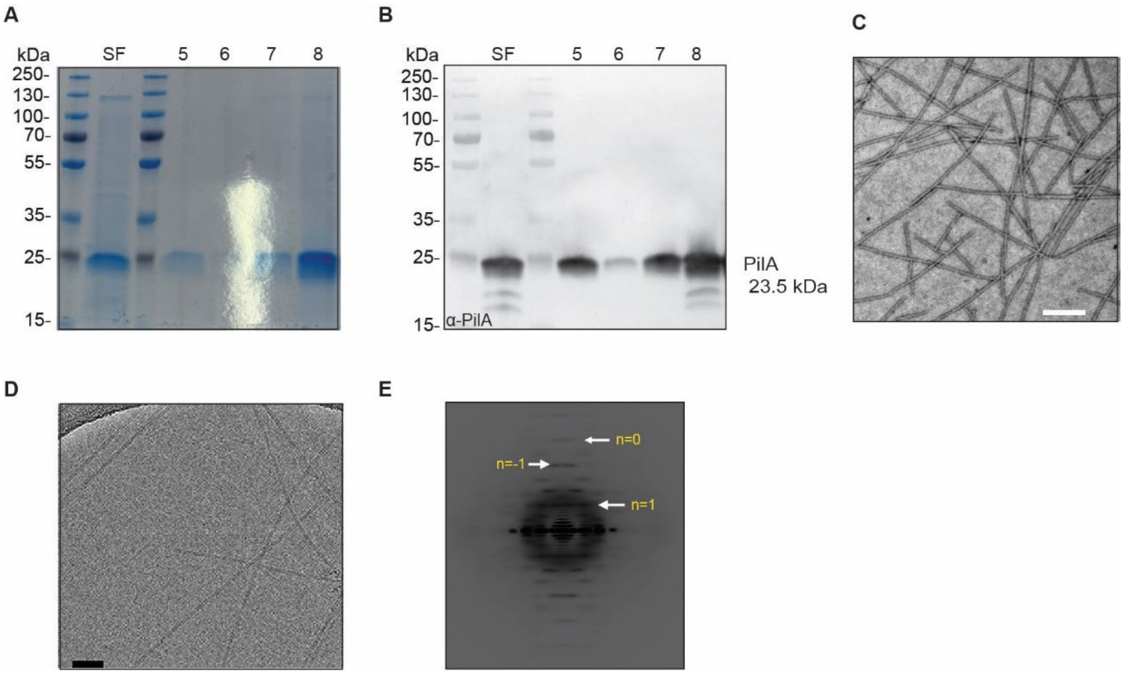


**Supplementary Fig. 2. Purification and structure elucidation of T4aP$^{Mx}$.**

**A-B.** T4aP$^{Mx}$ from Δ*pilT* cells were sheared-off and precipitated (sheared fraction = SF). The SF was further purified using sucrose-gradient centrifugation and fractions were collected. SF and PilA-containing fractions (5-8) were separated by SDS-PAGE and visualized by Coomassie protein staining (**A**) and probed with α-PilA antibodies (**B**). The calculated molecular mass of PilA and positions of molecular markers are indicated.

**C.** Representative micrograph of negatively stained T4aP$^{Mx}$. Scale bar, 200nm.

**D.** Representative cryo-EM micrograph of T4aP$^{Mx}$. Scale bar, 500nm.

**E.** Averaged power spectrum from T4aP$^{Mx}$.

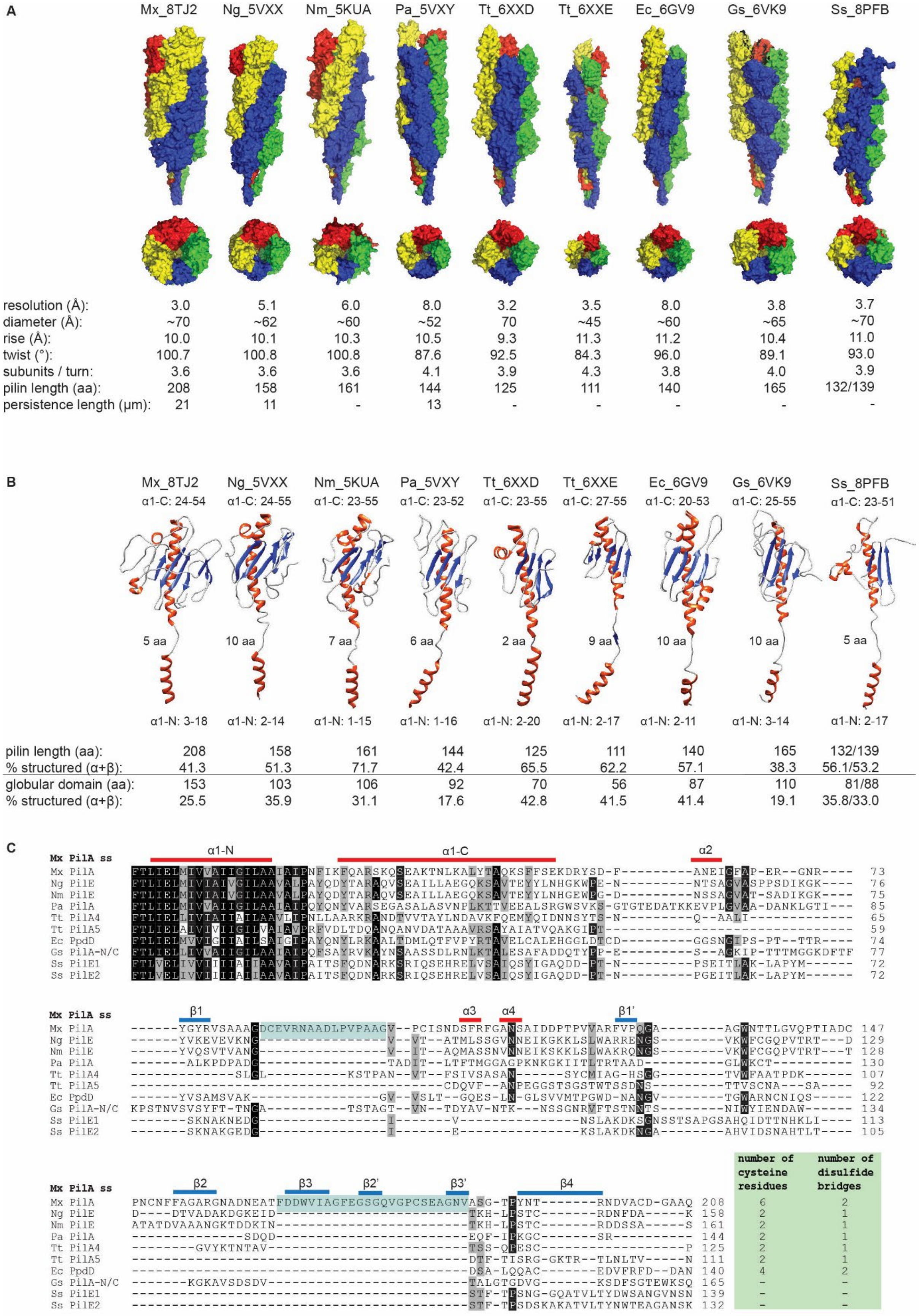

A
Mx_8TJ2 Ng_5VXX Nm_5KUA Pa_5VXY Tt_6XXD Tt_6XXE Ec_6GV9 Gs_6VK9 Ss_8PFB
resolution (Å): 3.0 5.1 6.0 8.0 3.2 3.5 8.0 3.8 3.7
diameter (Å): ~70 ~62 ~60 ~52 70 ~45 ~60 ~65 ~70
rise (Å): 10.0 10.1 10.3 10.5 9.3 11.3 11.2 10.4 11.0
twist (°): 100.7 100.8 100.8 87.6 92.5 84.3 96.0 89.1 93.0
subunits / turn: 3.6 3.6 3.6 4.1 3.9 4.3 3.8 4.0 3.9
pilin length (aa): 208 158 161 144 125 111 140 165 132/139
persistence length (µm): 21 11 - 13 - - - - -
B
Mx_8TJ2 Ng_5VXX Nm_5KUA Pa_5VXY Tt_6XXD Tt_6XXE Ec_6GV9 Gs_6VK9 Ss_8PFB
α1-C: 24-54 α1-C: 24-55 α1-C: 23-55 α1-C: 23-52 α1-C: 23-55 α1-C: 27-55 α1-C: 20-53 α1-C: 25-55 α1-C: 23-51
5 aa 10 aa 7 aa 6 aa 2 aa 9 aa 10 aa 10 aa 5 aa
α1-N: 3-18 α1-N: 2-14 α1-N: 1-15 α1-N: 1-16 α1-N: 2-20 α1-N: 2-17 α1-N: 2-11 α1-N: 3-14 α1-N: 2-17
pilin length (aa): 208 158 161 144 125 111 140 165 132/139
% structured (α+β): 41.3 51.3 71.7 42.4 65.5 62.2 57.1 38.3 56.1/53.2
globular domain (aa): 153 103 106 92 70 56 87 110 81/88
% structured (α+β): 25.5 35.9 31.1 17.6 42.8 41.5 41.4 19.1 35.8/33.0
C
Mx PilA ss α1-N α1-C α2
Mx PilA FTLIELMIVVAIIGILAAIAIPNFIKFQARSKQSEAKTNLKALYTAQKSFFSEKDRYSD-F---------ANEIGFAP-ER--GNR---- 73
Ng PilE FTLIELMIVIAIVGILAAVALPAYQDYTARAQVSEAILLAEGQKSAVTEYYLNHGKWPE-N---------NTSAGVASPPSDIKGK---- 76
Nm PilE FTLIELMIVIAIVGILAAVALPAYQDYTARAQVSEAILLAEGQKSAVTEYYLNHGEWPG-D---------NSSAGVAT-SADIKGK---- 75
Pa PilA FTLIELMIVVAIIGILAAIAIPQYQNYVARSEGASALASVNPLKTTVEEALSRGWSVKS-GTGTEDATKKEVPLGVAA-DANKLGTI--- 85
Tt PilA4 FTLIELLIVIAIIAILAAVLIPNLLAARKRANDTVVTAYLNDAVKFQEMYQIDNNSYTS-N---------Q--AALI------------ 65
Tt PilA5 FTLIELAIVIVIIGILVAIAVPRFVDLTDQANQANVDATAAAVRSAYAIATVQAKGIPT---------------------------------- 59
Ec PpdD FTLIELMVVIGIIAILSAIGIPAYQNYLRKAALTDMLQTFVPYRTAVELCALEHGGLDTCD---------GGSNGIPS-PT--TTR---- 74
Gs PilA-N/C FTLIELLIVVAIIGILAAIAIPQFSAYRVKAYNSAASSDLRNLKTALESAFADDQTYPP-E---------S--AGKIP-TTTMGGKDFTF 77
Ss PilE1 FTLVELIVVIIIIAITAAVAIPAITSFQDNARKSRIQSEHRELVSAIQSYIGAQDD-PT-N---------PSEITLAK-LAPYM------ 72
Ss PilE2 FTLVELIVVIIIIAIIAAVAIPAITSFQDNARKSRIQSEHRELVSAIQSYIGAQDD-PT-N---------PGEITLAK-LAPYM------ 72
Mx PilA ss β1 α3 α4 β1'
Mx PilA ------YGYRVSAAAGDCEVRNAADLPVPAAGV--PCISNDSFRFGANSAIDDPTPVVARFVPQGA---------AGWNTTLGVQPTIADC 147
Ng PilE ------YVKEVEVKNG----------------V--VT--ATMLSSGVNNEIKGKKLSLWARRENGS--------VKWFCGQPVTRT---D 129
Nm PilE ------YVQSVTVANG----------------V--IT--AQMASSNVNNEIKSKKLSLWAKRQNGS--------VKWFCGQPVTRT---T 128
Pa PilA -------ALKPDPADG----------------TADIT--LTFTMGGAGPKNKGKIITLTRTAAD-----------GLWKCT---------- 130
Tt PilA4 -------------SLGL----------KSTPAN--VT--FSIVSASAN------SYCMIAG-HSGG--------TVWFAATPDK------ 107
Tt PilA5 ----------------------------------------CDQVF--ANPEGGSTSGSTWTSSDNS----------TTVSCNA---SA---- 92
Ec PpdD ------YVSAMSVAK---------------GV--VSLT--GQES-LN-GLSVVMTPGWD-NANGV---------TGWARNCNIQS----- 122
Gs PilA-N/C KPSTNVSVSYFT-TNGA-----------TSTAGT--VN--TDYAV-NTK---NSSGNRVFTSTNNTS--------NIWYIENDAW------ 134
Ss PilE1 -------SKNAKNEDG----------------I-------V----------------NSLAKDKSGNSSTSAPGSAHQIDTTNHKLI---- 113
Ss PilE2 -------SKNAKGEDG----------------I-------E----------------KSLAKDKNGA---------AHVIDSNAHTLT---- 105
Mx PilA ss β2 β3 β2' β3' β4
number of cysteine residues | number of disulfide bridges
Mx PilA PNCNFFAGARGNADNEATFDDWVIAGFEGSGQVGPCSEAGNVASG-TPYNT-------RNDVACD-GAAQ 208 6 2
Ng PilE D----DTVADAKDGKEID--------------------------TKH-LPSTC-------RDNFDA-----K 158 2 1
Nm PilE ATATDVAAANGKTDDKIN--------------------------TKH-LPSTC-------RDDSSA-----S 161 2 1
Pa PilA --------------SDQD--------------------------EQF-IPKGC-------SR---------- 144 2 1
Tt PilA4 ---------GVYKTNTAV--------------------------TSS-QPESC--------------------P 125 2 1
Tt PilA5 ----------------------------------------------DTF-TISRG-GKTR--TLNLTV-----N 111 2 1
Ec PpdD ----------------------------------------------DSA-LQQAC-------EDVFRFD--DAN 140 4 2
Gs PilA-N/C -------KGKAVSDSDV---------------------------TALGTGDVG-------KSDFSGTEWKSQ 165 - -
Ss PilE1 ----------------------------------------------STF-TPSNG-GQATVLTYDWSANGVNSN 139 - -
Ss PilE2 ----------------------------------------------STF-TPSDSKAKATVLTYNWTEAGANSK 132 - -

**Supplementary Fig. 3. Comparisons of $T4aP^{Mx}$ and $PilA^{Mx}$ to other T4aP structures and T4a pilins.**

**A**. Helical arrangement of depicted T4aP with N and N+4 subunits shown in the same color in a side view (top row) and viewed from top ($2^{nd}$ row). T4aP characteristics (resolution, diameter, rise, twist, subunits/turn and pilin length were taken from this study (Mx_8TJ2) and previously solved T4aP structures (*N. gonorrhoeae*, Ng_5VXX (20); *N. meningitidis*, Nm_5KUA (21); *P. aeruginosa* PAK, Pa_5VXY (20); *T. thermophilus*, Tt_6XXD and Tt_6XXE (22); enterohemorrhagic *E. coli*, Ec_6GV9 (23), *G. sulfurreducens*, Gs_6VK9 (24); *S. sanguinis,* Ss_8PFB (25)). T4aP are shown as 12mers with the exception of Ss_8PFB, which is shown as a 9mer. Note that the major pilin of G. *sulfurreducens* is heterodimeric and composed of PilA-N (61 aa) and PilA-C (104 aa) (24), and the T4aP from *S. sanguinis* is heteropolymeric and composed of two very similar major pilins PilE1 and PilE2 (139 and 132 aa) (25).The last row indicates persistence length as measured and depicted in Fig. S5A.

**B**. Ribbon representation of $PilA^{Mx}$ and depicted pilins from previously solved T4aP structures as in **A** with helical elements (α) shown in red, β-stranded elements (β) shown in blue and less-structured areas (loops) in grey. The first and last residues of the helices of α1-N and α1-C are shown as well as the number of residues (aa) in the melted region. Pilin characteristics (length of pilin and globular domain (aa) and % structured (α+β) were taken from this study and previously solved T4aP structures as in **A**.

**C.** Multiple sequence alignment of $PilA^{Mx}$ and pilins from previously solved T4aP structures as in **A**. The top row indicates the structural elements of $PilA^{Mx}$ as in Fig. 3B. The blue shaded areas indicate extra residues of $PilA^{Mx}$ in two areas (between β1 and α3 and between β2 and β4). Residues on black are conserved in ≥60% of the proteins, and residues on gray are similar in ≥60% of the proteins. The green shaded box at the end of the alignment depicts numbers of cysteine residues and disulfide bridges in the aligned T4a pilins.

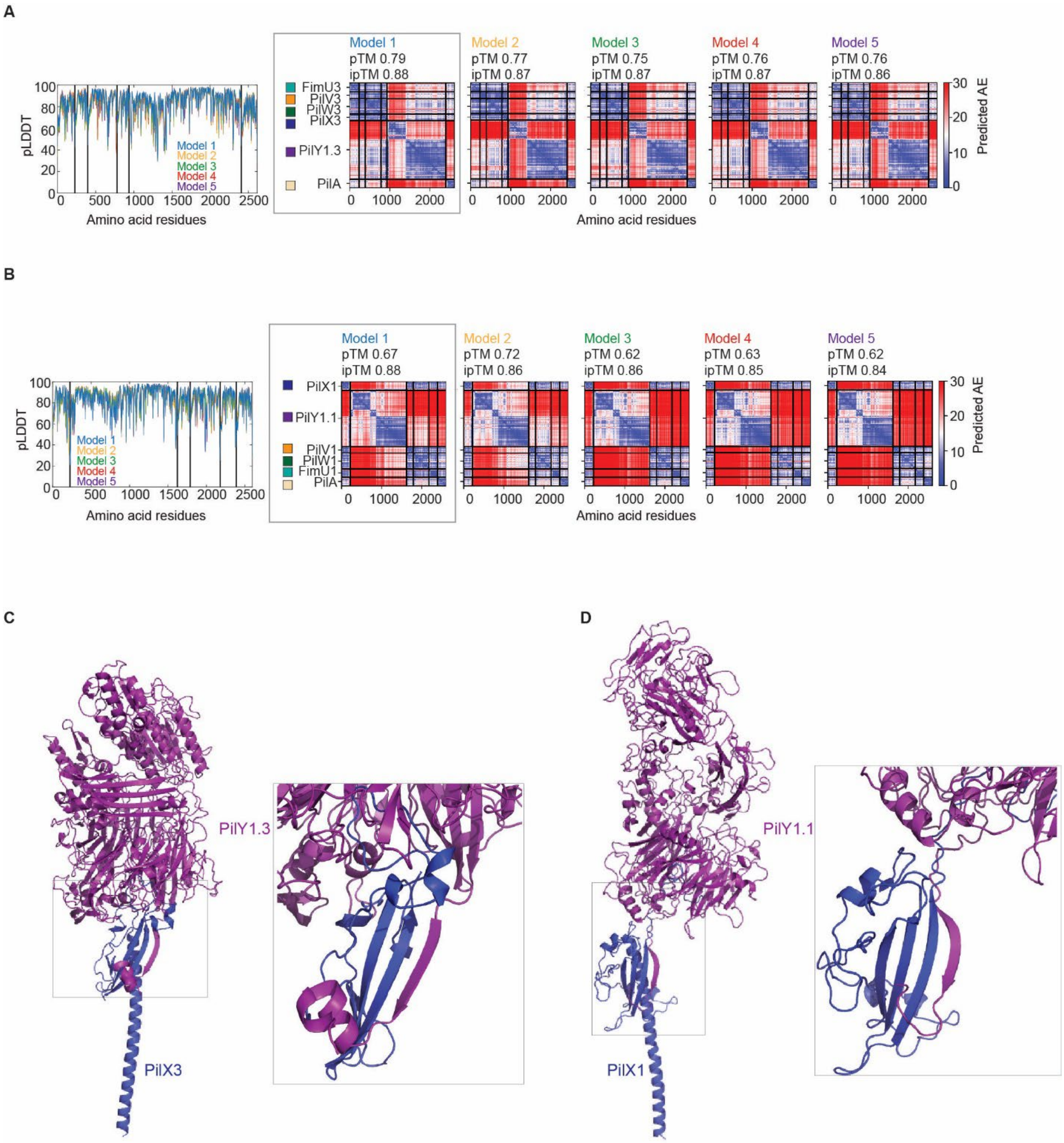


**Supplementary Fig. 4. AlphaFold-Multimer models of two different tip complexes of *M. xanthus*.**

**A**. Plots generated by AlphaFold-Multimer for the complex of cluster_3 proteins (FimU3, PilV3, PilW3, PilV3, and PilY1.3) and PilA with pLDDT (left) and pAE plots (right) for five models of the indicated proteins as predicted by AlphaFold-Multimer. The five models were generated and ranked according to their pTM and iPTM scores, which are shown on top of the individual models. The highest ranked model is marked by a grey box. Models 1-5 in the pLDDT (left) and

pAE plots (right) are shown in the same colors. The color code of the six proteins is as in Fig. 4A.

**B**. Plots generated by AlphaFold-Multimer for the complex of cluster_1 proteins (FimU1, PilV1, PilW1, PilX1, and PilY1.1) and PilA as in **A**. The color code of the six proteins is as in Fig. 4B.

**C**. Predicted protein-protein interaction by β-strand addition between PilY1.3 (purple) and PilX3 (blue) by the AlphaFold-Multimer model. The boxed area shows the area of the β-strand addition without α1 of PilX3.

**D.** Predicted protein-protein interaction by β-strand addition between PilY1.1 (purple) and PilX1 (blue) by the AlphaFold model of cluster_1 proteins. The boxed area shows the area of the β-strand addition without α1 of PilX1.

**A-D.** The sequences of the mature minor pilins, and PilY1 proteins without their signal peptides as reported earlier (15), were used for generating the models.

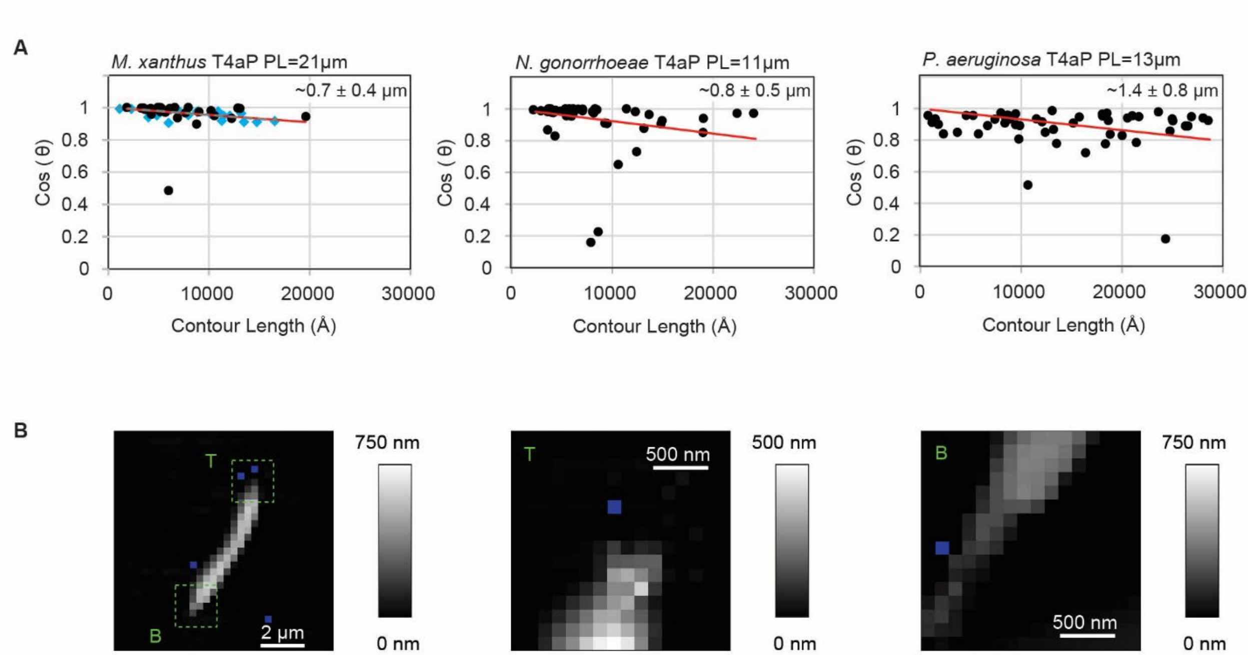


**Supplementary Fig. 5. Persistence length analyses of T4aP and AFM-MS analyses of *M. xanthus* cells.**

**A** The cosine of the bending angle (theta) of a T4aP segment is plotted against the contour length of the segment (Å). PL (µm) is calculated from the slope (grey & red lines) of the linear trendline. Negative staining micrographs of purified T4aP from *M. xanthus* (left;T4aP from WT cells as blue diamonds, slope as dashed grey line; T4aP from ∆*pilT* cells as black circles, slope as red line as in Fig. S2C), T4aP from *N. gonorrhoeae* (center), and T4aP from *P. aeruginosa* PAK (right) were used for this analysis. The mean contour length ± standard deviation (STDEV) is shown in µm in the upper right corner.

**B.** Overlaid adhesion and height images of *M. xanthus* ∆*pilA* cells obtained using the same force spectroscopy parameters used for WT (Fig. 5C). Left: Representative cell, middle: zoom of the pole at the top (T) (16 × 16 pixels), right: zoom of the pole at the bottom (B) (bottom, 16 × 16 pixels). Signatures resembling nanosprings were detected sporadically. Plateau signatures were not detected.

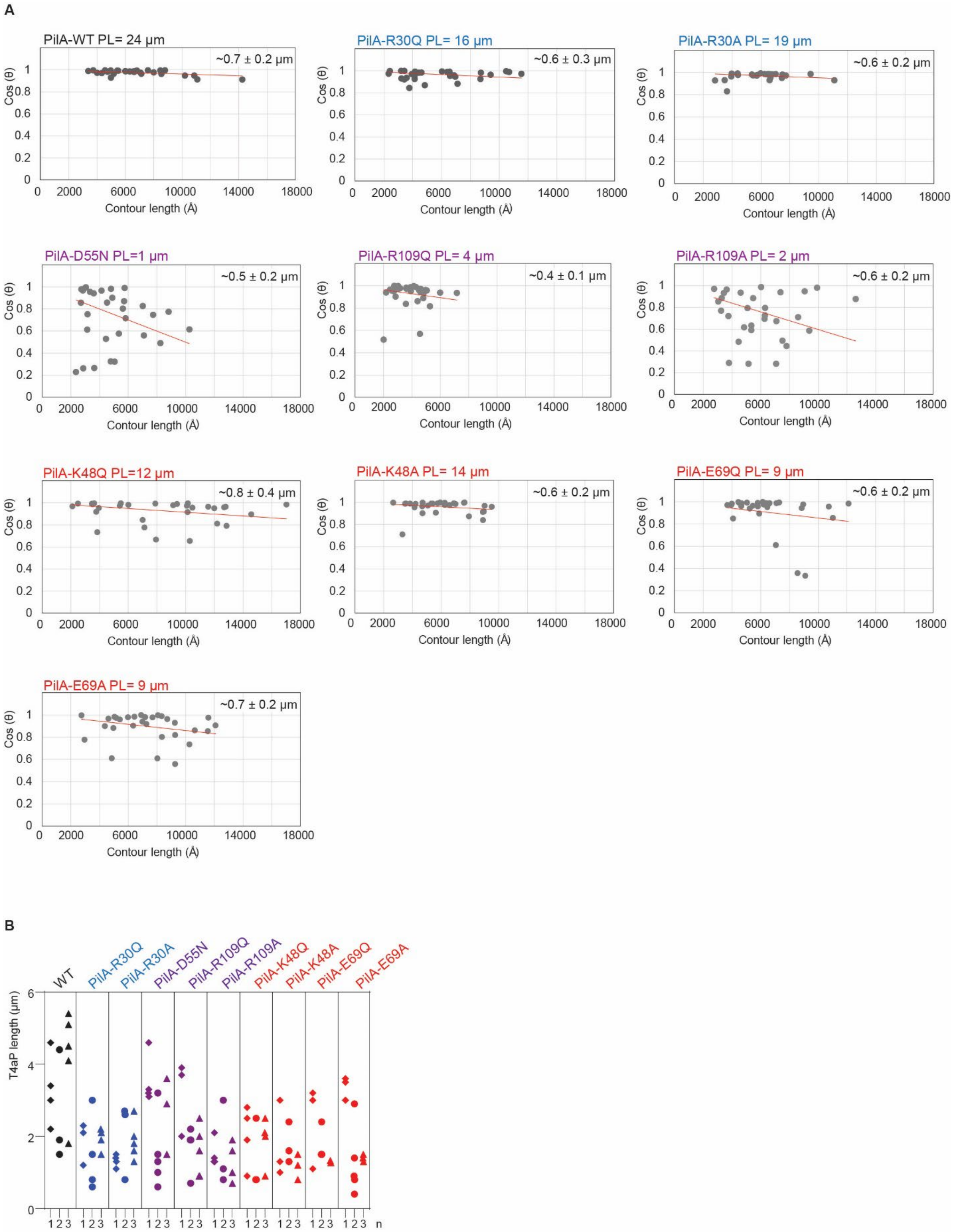


**Supplementary Fig. 6. Persistence length and T4aP length analyses.**

**A.** The cosine of the bending angle (theta) of a T4aP segment is plotted against the contour

length of the segment (Å). PL (µm) is calculated from the slope of the linear trendline as in Fig. S5A. The mean contour length ± standard deviation (STDEV) is shown in µm in the upper right corner. Negative staining micrographs of sheared pili from Δ*pilT* strains expressing $PilA^{WT}$ and indicated PilA variants as shown in Fig. 5F were used for this analysis.

**B**. WT and PilA-variants were imaged by TEM and negative staining electron micrographs of cells from indicated strains were analyzed. For each indicated strain, the length of T4aP in µm from three cells was measured (diamond, circle, star).

**Supplementary Table 1. Phylogenetic distribution of large major pilins on the order level.**

| phyla (proteobacteria as classes) | No* | Order | *Species* | locus tag | length* (aa) |
|---|---|---|---|---|---|
| Proteobacteria-Gamma | 22 | Acidoferrobacterales | *Acidiferrobacter sp. SPIII_3* | acii_C4901_15195 | 215 |
| | | | | acii_C4901_15470 | 216 |
| | | Moraxellales | *Acinetobacter lanii* | alj_G8D99_13855 | 172 |
| | | | *Acinetobacter ursingii* | aug_URS_1380 | 166 |
| | | | *Acinetobacter wanghuae* | awg_GFH30_11740 | 179 |
| | | Thiotrichales | *Francisella adeliensis* | fad_CDH04_05240 | 258 |
| | | | *Francisella philomiragia O#319-036* | fpm_LA56_551 | 297 |
| | | | *Francisella philomiragia GA01-2794* | fpz_LA55_1175 | 268 |
| | | | *Francisella uliginis* | frx_F7310_06235 | 221 |
| | | | *Francisella tularensis subsp. tularensis FSC198* | ftf_FTF0888c | 189 |
| | | Chromatiales | *Halothiobacillus neapolitanus* | hna_Hneap_0014 | 166 |
| | | Cellvibrionales | *Microbulbifer aggregans* | micc_AUP74_03352 | 167 |
| | | | | micc_AUP74_03354 | 172 |
| | | Pseudomonadales | *Pseudomonas aeruginosa UCBPP-PA14* | pau_PA14_58730 | 173 |
| | | Oceanopspirillales | *Reinekea forsetii* | rfo_REIFOR_02656 | 181 |
| | | Nevskiales | *Steroidobacter denitrificans* | sdf_ACG33_12010 | 182 |
| | | Xanthomonadales | *Xylella fastidiosa 9a5c* | xfa_XF_0538_ 180 | 180 |
| | | | | xfa_XF_0539_ 180 | 180 |
| | | | *Xylella fastidiosa subsp. fastidiosa GB514* | xff_XFLM_11165 | 173 |
| | | | *Xylella fastidiosa subsp. sandyi Ann-1* | xfs_D934_07230 | 182 |
| | | | *Xylella taiwanensis* | xtw_AB672_05610 | 182 |
| | | | | xtw_AB672_11770 | 174 |
| Proteobacteria-Beta | 75 | Burkholderiales | *Achromobacter sp. B7* | achb_DVB37_08520 | 189 |
| | | | *Achromobacter spanius* | asw_CVS48_14600 | 189 |
| | | | *Achromobacter xylosoxidans A8* | axy_AXYL_02161 | 190 |
| | | | *Acidovorax carolinensis NA2* | acid_CBP33_18415 | 180 |
| | | | | acid_CBP33_18420 | 183 |
| | | | *Acidovorax carolinensis NA3* | acin_CBP34_18600 | 169 |
| | | | *Acidovorax ebreus* | dia_Dtpsy_3385 | 185 |
| | | | | dia_Dtpsy_3387 | 188 |
| | | | *Acidovorax sp. 1608163* | acio_EAG14_00085 | 166 |
| | | | | acio_EAG14_00090 | 168 |
| | | | *Acidovorax sp. JS42* | ajs_Ajs_4040 | 170 |
| | | | *Acidovorax sp. KKS102* | ack_C380_23265 | 171 |
| | | | *Alicycliphilus denitrificans BC* | adn_Alide_4260 | 181 |
| | | | *Alicycliphilus denitrificans K601* | adk_Alide2_4600 | 184 |

| | | | | | |
|---|---|---|---|---|---|
| | | | *Bordetella avium* | bav_BAV2358 | 181 |
| | | | *Bordetella bronchialis* | bbro_BAU06_08730 | 189 |
| | | | *Bordetella bronchiseptica 253* | bbh_BN112_0995 | 189 |
| | | | *Bordetella flabilis* | bfz_BAU07_17430 | 189 |
| | | | *Bordetella genomosp. 13* | bgm_CAL15_10280 | 189 |
| | | | *Bordetella hinzii* | bhz_ACR54_03455 | 180 |
| | | | *Bordetella petrii* | bpt_Bpet2000 | 189 |
| | | | *Bordetella sp. H567* | boh_AKI39_08505 | 189 |
| | | | *Bordetella trematum* | btrm_SAMEA390648 | 181 |
| | | | *Burkholderia cenocepacia DDS 22E-1* | bcen_DM39_2733 | 167 |
| | | | *Burkholderia cepacia DDS 7H-2* | bced_DM42_2426 | 167 |
| | | | *Burkholderia cepacia GG4* | bct_GEM_0791 | 167 |
| | | | *Burkholderia dolosa* | bdl_AK34_443 | 167 |
| | | | *Burkholderia glumae BGR1* | bgl_bglu_1g29910 | 167 |
| | | | *Burkholderia multivorans DDS 15A-1* | bmk_DM80_2324_ | 167 |
| | | | *Burkholderia sp. PAMC 26561* | bum_AXG89_08900 | 167 |
| | | | *Burkholderia stabilis* | bstl_BBJ41_15555 | 167 |
| | | | *Burkholderia stagnalis* | bstg_WT74_14030 | 167 |
| | | | *Burkholderia vietnamiensis G4* | bvi_Bcep1808_2152 | 240 |
| | | | | bvi_Bcep1808_2158 | 279 |
| | | | *Burkholderiales bacterium GJ-E10* | bbag_E1O_23150 | 169 |
| | | | *Caballeronia insecticola* | buo_BRPE64_ACDS 04770 | 167 |
| | | | *Caldimonas brevitalea* | pbh_AAW51_0098 | 167 |
| | | | *Castellaniella defragrans* | cdn_BN940_08181 | 173 |
| | | | *Comamonas serinivorans* | cser_CCO03_18970 | 166 |
| | | | | cser_CCO03_18975 | 168 |
| | | | *Comamonas testosteroni TK102* | ctes_O987_14600 | 170 |
| | | | *Delftia lacustris* | dla_I6G47_20305 | 187 |
| | | | *Delftia sp. Cs1-4* | del_DelCs14_5662 | 166 |
| | | | *Delftia tsuruhatensis* | dts_BI380_20350 | 167 |
| | | | *Diaphorobacter ruginosibacter* | drg_H9K76_21515 | 177 |
| | | | *Diaphorobacter sp. HDW4A* | dih_G7047_25905 | 188 |
| | | | *Hydrogenophaga pseudoflava* | hpse_HPF_22800 | 172 |
| | | | | hpse_HPF_22805 | 175 |
| | | | *Hydrogenophaga sp. LPB0072* | hyl_LPB072_22445 | 180 |
| | | | | hyl_LPB072_22450 | 181 |
| | | | *Massilia oculi* | mtim_DIR46_18135 | 175 |
| | | | *Massilia sp. YMA4* | masy_DPH57_1752 5 | 166 |
| | | | *Mycetohabitans rhizoxinica* | brh_RBRH_01328 | 166 |

| | | | | | |
|---|---|---|---|---|---|
| | | | *Ottowia sp. oral taxon 894* | oto_ADJ79_06795 | 168 |
| | | | *Ottowia sp. oral taxon 894* | oto_ADJ79_06800 | 170 |
| | | | *Paenalcaligenes hominis* | phn_PAEH1_04210 | 168 |
| | | | *Paraburkholderia tropica* | ptro_G5S35_11920 | 167 |
| | | | *Pigmentiphaga aceris* | pacr_FXN63_19155 | 175 |
| | | | *Pigmentiphaga sp. H8* | pig_EGT29_07315 | 180 |
| | | | *Polaromonas naphthalenivorans* | pna_Pnap_4333 | 178 |
| | | | *Pulveribacter suum* | melm_C7H73_02965 | 181 |
| | | | *Pusillimonas sp. DMV24BSW_D* | pud_G9Q38_12345 | 171 |
| | | | *Pusillimonas sp. T7-7* | put_PT7_1417 | 176 |
| | | | *Rhodoferax sediminis Gr-4* | rhg_EXZ61_01095 | 168 |
| | | | *Simplicispira suum* | simp_C6571_08580 | 169 |
| | | | *Verminephrobacter eiseniae* | vei_Veis_4782 | 167 |
| | | Neisseriales | *Neisseria bacilliformis* | nbc_H3L91_00080 | 172 |
| | | | | nbc_H3L91_00085 | 173 |
| | | | | nbc_H3L91_00090 | 173 |
| | | | *Neisseria elongata* | nel_NELON_01840 | 167 |
| | | | | nel_NELON_01845 | 181 |
| | | | *Neisseria zalophi* | nzl_D0T92_10455 | 175 |
| | | | | nzl_D0T92_10460 | 167 |
| | | | *Simonsiella muelleri* | smur_BWP33_11975 | 166 |
| | | Nitrosomonadales | *Sideroxydans lithotrophicus* | slt_Slit_0538 | 172 |
| Firmicutes | 25 | Bacillales | *Anoxybacillus flavithermus* | afl_Aflv_0627 | 168 |
| | | | *Bacillus selenitireducens* | bse_Bsel_0234 | 191 |
| | | | *Geobacillus sp. LC300* | gel_IB49_04990 | 168 |
| | | | *Geobacillus stearothermophilus* | gse_GT50_04575 | 168 |
| | | | *Geobacillus subterraneus* | gsr_GS3922_03060 | 169 |
| | | Erysipelotrichales | *Intestinibaculum porci* | ebm_SG0102_16640 | 167 |
| | | Eubacteriales | *Acetivibrio saccincola* | hsc_HVS_10140 | 192 |
| | | | *Anaerostipes rhamnosivorans* | arf_AR1Y2_1992 | 280 |
| | | | *Clostridioides difficile 630* | cdf_CD630_35080 | 178 |
| | | | *Clostridium kluyveri DSM 555* | ckl_CKL_1112 | 166 |
| | | | *Clostridium perfringens 13* | cpe_CPE2284 | 170 |
| | | | *Eubacterium maltosivorans* | emt_CPZ25_010940 | 179 |
| | | | *Eubacterium siraeum 70/3* | esu_EUS_24460 | 210 |
| | | | | esu_EUS_24480 | 217 |
| | | | *Eubacterium siraeum V10Sc8a* | esr_ES1_06510 | 203 |
| | | | | esr_ES1_06520 | 210 |
| | | | *Geosporobacter ferrireducens* | gfe_Gferi_00415 | 234 |

| | | | | | |
|---|---|---|---|---|---|
| | | | *Hungateiclostridium thermocellum ATCC 27405* | cth_Cthe_1103 | 180 |
| | | | | cth_Cthe_1104 | 166 |
| | | | *Hungateiclostridium thermocellum DSM 1313* | ctx_Clo1313_1110 | 178 |
| | | | *Ruminococcus albus* | ral_Rumal_3060 | 237 |
| | | | *Ruminococcus bicirculans* | rus_RBI_I01475 | 169 |
| | | | *Ruminococcus champanellensis* | rch_RUM_03620 | 195 |
| | | | | rch_RUM_16890 | 173 |
| | | | *Thermoclostridium stercorarium DSM 8532* | css_Cst_c12640 | 173 |
| Cyanobacteria | 29 | Chroococcales | *Crocosphaera subtropica* | cyt_cce_2840 | 180 |
| | | | *Gloeothece citriformis* | cyc_PCC7424_0260 | 167 |
| | | Gloeobacterales | *Gloeobacter kilaueensis* | glj_GKIL_0446 | 185 |
| | | | | glj_GKIL_4335 | 172 |
| | | | *Gloeobacter violaceus* | gvi_gll2255 | 171 |
| | | | | gvi_glr3426 | 186 |
| | | Nostocales | *Calothrix sp. PCC 6303* | calt_Cal6303_362 | 183 |
| | | | *Cylindrospermum stagnale* | csg_Cylst_2769 | 174 |
| | | | *Nostoc flagelliforme* | nfl_COO91_06898 | 168 |
| | | Oscillatoriales | *Geitlerinema sp. PCC 7407* | gei_GEI7407_3664 | 169 |
| | | | *Microcoleus sp. PCC 7113* | mic_Mic7113_5040 | 174 |
| | | | *Moorea producens* | mpro_BJP34_19960 | 175 |
| | | Pseudoanabaenales | *Leptolyngbya sp. NIES-3755* | len_LEP3755_39030 | 176 |
| | | | *Leptolyngbya sp. PCC 7376* | lep_Lepto7376 | 243 |
| | | | *Pseudanabaena sp. ABRG5-3* | pser_ABRG53_0283 | 172 |
| | | | *Pseudanabaena sp. PCC 7367* | pseu_Pse7367_2053 | 177 |
| | | | | pseu_Pse7367_2054 | 166 |
| | | | *Halomicronema hongdechloris* | hhg_XM38_026330 | 173 |
| | | | *Thermosynechococcus elongatus* | tel_tlr0682_ 172 | 172 |
| | | | *Thermosynechococcus sp. NK55* | thn_NK55_01305 | 172 |
| | | Synechococcales | *Chamaesiphon minutus* | cmp_Cha6605_2755 | 168 |
| | | | *Cyanobium gracile* | cgc_Cyagr_1414 | 169 |
| | | | *Prochlorococcus marinus MIT 9211* | pmj_P9211_15291 | 171 |
| | | | *Prochlorococcus marinus MIT 9301* | pmg_P9301_07011 | 175 |
| | | | *Prochlorococcus marinus MIT 9312* | pmi_PMT9312_1201 | 177 |
| | | | *Synechococcus sp. PCC 7003* | syl_AWQ21_14105 | 173 |
| | | | *Synechococcus sp. PCC 73109* | syv_AWQ23_13975 | 212 |
| | | | *Synechococcus sp. PCC 7502* | synp_Syn7502_00935 | 198 |
| | | | *Synechococcus sp. PCC 7002* | syp_SYNPCC7002_A2804 | 170 |

| | | | | | |
|---|---|---|---|---|---|
| Actinobacteria | 4 | Bifidobacteriales | *Bifidobacterium thermophilum* | btp_D805_0701 | 187 |
| | | Corynebacteriales | *Corynebacterium callunae* | ccn_H924_06030 | 224 |
| | | Eggerthellales | *Gordonibacter pamelaeae* | gpa_GPA_11520 | 240 |
| | | Euzebyales | *Euzebya pacifica DY32-46* | euz_DVS28_b0477 | 227 |
| Deinococcus-Thermus | 1 | Thermales | *Thermus sp. CCB_US3_UF1* | thc_TCCBUS3UF1_3990 | 176 |
| Thermodesulfo-bacteria | 7 | Desulfobulbales | *Desulfobulbus oralis* | deo_CAY53_11360 | 200 |
| | | | *Desulfurivibrio alkaliphilus* | dak_DaAHT2_2283 | 182 |
| | | Desulfuromonadales | *Desulfuromonas soudanensis* | des_DSOUD_2157 | 193 |
| | | | *Pelobacter carbinolicus* | pca_Pcar_2143 | 170 |
| | | Geobacterales | *Geobacter daltonii FRC-32* | geo_Geob_3369 | 208 |
| | | | *Geobacter uraniireducens* | gur_Gura_2677 | 193 |
| | | Desulfomonilales | *Desulfomonile tiedjei* | dti_Desti_4024 | 204 |
| Myxococcota | 39 | Myxococcales | *Anaeromyxobacter dehalogenans 2CP-1* | acp_A2cp1_0669 | 188 |
| | | | *Anaeromyxobacter sp. Fw109-5* | afw_Anae109_0680 | 191 |
| | | | *Archangium gephyra* | age_AA314_07645 | 202 |
| | | | | age_AA314_08283 | 204 |
| | | | *Chondromyces crocatus* | ccro_CMC5_052100 | 182 |
| | | | | ccro_CMC5_052110 | 188 |
| | | | | ccro_CMC5_052120 | 193 |
| | | | *Corallococcus coralloides* | ccx_COCOR_06280 | 198 |
| | | | *Corallococcus macrosporus* | mfu_LILAB_36405 | 203 |
| | | | *Cystobacter fuscus* | cfus_CYFUS_00131 | 202 |
| | | | | cfus_CYFUS_00190 | 202 |
| | | | | cfus_CYFUS_00751 | 201 |
| | | | *Haliangium ochraceum* | hoh_Hoch_4154 | 175 |
| | | | | hoh_Hoch_4155 | 177 |
| | | | *Labilithrix luteola* | llu_AKJ09_08823 | 185 |
| | | | | llu_AKJ09_08824 | 166 |
| | | | *Melittangium boletus* | mbd_MEBOL_001002 | 201 |
| | | | | mbd_MEBOL_001435 | 203 |
| | | | *Minicystis rosea* | mrm_A7982_03796 | 187 |
| | | | | mrm_A7982_03797 | 173 |
| | | | *Myxococcus fulvus* | mfb_MFUL124B02 | 183 |
| | | | *Myxococcus hansupus* | mym_A176_001126 | 217 |
| | | | *Myxococcus macrosporus* | mmas_MYMAC_00557 | 205 |
| | | | *Myxococcus stipitatus* | msd_MYSTI_06366 | 184 |
| | | | *Myxococcus xanthus* | mxa_MXAN_5783 | 208 |
| | | | *Sandaracinus amylolyticus* | samy_DB32_004027 | 176 |
| | | | | samy_DB32_004028 | 176 |

| | | | | | |
|---|---|---|---|---|---|
| | | | *Sorangium cellulosum So ce56* | scl_sce4273 | 179 |
| | | | | scl_sce4274 | 192 |
| | | | | scl_sce4275 | 192 |
| | | | *Sorangium cellulosum So0157-2* | scu_SCE1572_2584 | 178 |
| | | | | scu_SCE1572_2585 | 193 |
| | | | *Stigmatella aurantiaca* | sur_STAUR_0003 | 202 |
| | | | | sur_STAUR_0004 | 203 |
| | | | | sur_STAUR_1125 | 203 |
| | | | | sur_STAUR_6449 | 205 |
| | | | | sur_STAUR_6450 | 204 |
| | | | | sur_STAUR_6924 | 204 |
| | | | *Vulgatibacter incomptus* | vin_AKJ08_0698 | 171 |
| Verrucomicrobiota | 4 | Opitutales | *Nibricoccus aquaticus HZ-65* | vbh_CMV30_12470 | 166 |
| | | | | vbh_CMV30_16935 | 210 |
| | | Verrucomicrobiales | *Luteolibacter luteus* | luo_HHL09_15680 | 188 |
| | | | *Roseimicrobium sp. ORNL1* | roo_G5S37_24550 | 223 |
| Aquificae | 1 | Desulfurobacteriales | *Thermovibrio ammonificans* | tam_Theam_1352 | 185 |
| Acidobacteriota | 1 | Acidobacteriales | *Koribacter versatilis* | aba_Acid345_2448 | 180 |
| Proteobacteria-Acidithiobacilli | 1 | Acidithiobacillaes | *Acidithiobacillus ferrivorans* | afi_Acife_1976 | 190 |
| Bdellovibrionota | 11 | Bacteriovoracales | *Bacteriovorax stolpii* | bsto_C0V70_00655 | 170 |
| | | | *Halobacteriovorax marinus* | bmx_BMS_0122 | 183 |
| | | | *Halobacteriovorax sp. BALOs_7* | hax_BALOs_0146 | 176 |
| | | Bdellovibrionales | *Bdellovibrio bacteriovorus 109J* | bbac_EP01_16385 | 180 |
| | | | *Bdellovibrio bacteriovorus HD100* | bba_Bd1290 | 179 |
| | | | *Bdellovibrio bacteriovorus Tiberius* | bbat_Bdt_1269 | 186 |
| | | | *Bdellovibrio bacteriovorus W* | bbw_BDW_04435 | 189 |
| | | | *Bdellovibrio exovorus* | bex_A11Q_1638 | 181 |
| | | | *Bdellovibrio sp. NC01* | bdc_DOE51_05870 | 186 |
| | | | *Bdellovibrio sp. qaytius* | bdq_CIK05_05425 | 204 |
| | | | *Bdellovibrio sp. ZAP7* | bdz_DOM22_13925 | 177 |
| Proteobacteria-Alpha | 2 | no rank | *Alpha proteobacterium HIMB5* | apm_HIMB5_00008 1 | 195 |
| | | | *Puniceispirillum marinum (Candidatus)* | apb_SAR116_2527 | 188 |
| Nitrospirota | 3 | Nitrospirales | *Nitrospira defluvii* | nde_NIDE3438 | 169 |
| | | | | nde_NIDE3439 | 166 |
| | | | *Nitrospira inopinata (Candidatus)* | nio_NITINOP_2648 | 196 |
| Armatimonadota | 1 | Chthonomonadales | *Chthonomonas calidirosea* | ccz_CCALI_00192 | 176 |

#The number represents the total number of large pilins in the corresponding phyla or probacterial class, respectively. *The number represents the mature major pilin length in aa.

**Supplementary Table 2. Number of major pilins and large major pilins in the category other**

| | phyla (proteobacteria shown as class) * | n | ≥166 aa | % of n |
|---|---|---|---|---|
| | **all** | **1955** | **226** | **11.6** |
| | other# | 146 | 24 | 10.6 |
| VER | Verrumicrobia | 17 | 4 | 23.5 |
| AQU | Aquificae | 17 | 1 | 5.8 |
| ACI | Acidobacteria | 16 | 1 | 6.2 |
| CHL | Chloroflexi | 15 | | |
| FUS | Fusobacteria | 14 | | |
| P-T | Proteobacteria-Acidithiobacilli | 12 | 1 | 0.4 |
| BDE | Bdellovibrionota | 11 | 11 | 100 |
| PLA | Planctomycetes | 9 | | |
| CAM | Campylobacterota | 9 | | |
| P-A | Proteobacteria-Alpha | 6 | 2 | 33.3 |
| NIT | Nitrospirae | 6 | 3 | 50.0 |
| GEM | Gemmatimonadetes | 5 | | |
| DES | Desferribacteres | 4 | | |
| ARM | Armatimonadetes | 3 | 1 | 33.3 |
| TEN | Tenericutes | 2 | | |

*Phyla are shaded in green and proteobacterial classes are shaded in yellow.
#The category other includes phyla or proteobacterial classes with only small numbers of pilins (each less than 1 % of all). Please note that in Fig. 1A this category is presented in two columns called “other” and Bdellovibrionota.

261 **Supplementary Table 3. *M. xanthus* and *E. coli* strains used in this work.**

| Strain | Description/Genotype[1] | Reference or source |
|---|---|---|
| ***M. xanthus*** | | |
| DK1622 | wildtype | (17) |
| DK10410 | Δ*pilA* (ΔMXAN_5783) | (26) |
| DK10409 | Δ*pilT* (ΔMXAN_5787) | (26) |
| SA11429 | *pilA*::*pilA*-R30Q (pMAT473) | This study |
| SA11431 | *pilA*::*pilA*-R30A (pMAT474) | This study |
| SA11435 | *pilA*::*pilA*-K37Q (pMAT476) | This study |
| SA11437 | *pilA*::*pilA*-K37A (pMAT477) | This study |
| SA11439 | *pilA*::*pilA*- E53Q (pMAT479) | This study |
| SA11441 | *pilA*::*pilA*- E53A (pMAT480) | This study |
| SA11451 | *pilA*::*pilA*-D55N (pMAT485) | This study |
| SA11453 | *pilA*::*pilA*-D55A (pMAT486) | This study |
| SA11457 | *pilA*::*pilA*-R73Q (pMAT488) | This study |
| SA11459 | *pilA*::*pilA*-R73A (pMAT489) | This study |
| SA11463 | *pilA*::*pilA*-R109Q (pMAT491) | This study |
| SA11491 | *pilA*::*pilA*-R109A (pMAT492) | This study |
| SA11469 | *pilA*::*pilA*-K48Q (pMAT494) | This study |
| SA11471 | *pilA*::*pilA*-K48A (pMAT495) | This study |
| SA11475 | *pilA*::*pilA*-E69Q (pMAT497) | This study |
| SA11477 | *pilA*::*pilA*-E69A (pMAT498) | This study |
| SA11497 | *pilA*::*pilA*-R70Q (pMAT528) | This study |
| SA11499 | *pilA*::*pilA*-R70A (pMAT505) | This study |
| SA11430 | *pilA*::*pilA*-R30Q (pMAT473) | This study |
| SA11432 | Δ*pilT*; *pilA*::*pilA*-R30A (pMAT474) | This study |
| SA11436 | Δ*pilT*; *pilA*::*pilA*-K37Q (pMAT476) | This study |

| | | |
|---|---|---|
| SA11485 | Δ*pilT*; *pilA*::*pilA*-K37A (pMAT477) | This study |
| SA11440 | Δ*pilT*; *pilA*::*pilA*- E53Q (pMAT479) | This study |
| SA11442 | Δ*pilT*; *pilA*::*pilA*- E53A (pMAT480) | This study |
| SA11452 | Δ*pilT*; *pilA*::*pilA*-D55N (pMAT485) | This study |
| SA11454 | Δ*pilT*; *pilA*::*pilA*-D55A (pMAT486) | This study |
| SA11458 | Δ*pilT*; *pilA*::*pilA*-R73Q (pMAT488) | This study |
| SA11460 | Δ*pilT*; *pilA*::*pilA*-R73A (pMAT489) | This study |
| SA11464 | Δ*pilT*; *pilA*::*pilA*-R109Q (pMAT491) | This study |
| SA11466 | Δ*pilT*; *pilA*::*pilA*-R109A (pMAT492) | This study |
| SA11470 | Δ*pilT*; *pilA*::*pilA*-K48Q (pMAT494) | This study |
| SA11472 | Δ*pilT*; *pilA*::*pilA*-K48A (pMAT495) | This study |
| SA11476 | Δ*pilT*; *pilA*::*pilA*-E69Q (pMAT497) | This study |
| SA11478 | Δ*pilT*; *pilA*::*pilA*-E69A (pMAT498) | This study |
| SA12322 | Δ*pilT*; *pilA*::*pilA*-R70Q (pMAT528) | This study |
| SA12300 | Δ*pilT*; *pilA*::*pilA*-R70A (pMAT505) | This study |
| ***E. coli*** | | |
| NEB® Turbo | F' *proA*$^+$*B*$^+$ *lacI*$^q$ Δ*lacZM15 / fhuA2* Δ*(lac-proAB) glnV galK16 galE15 R(zgb-210::Tn10)*Tet$^S$ *endA1 thi-1* Δ*(hsdS-mcrB)5* | New England Biolabs |

[1] Plasmids used for construction of *pilA*-variants at the endogenous locus are indicated in brackets.

**Supplementary Table 4. Plasmids used in this work.**

| **Plasmids** | | |
|---|---|---|
| pBJ114 | *galK* containing vector for generation of in-frame deletions in *M. xanthus*, Kan$^R$ | (27) |
| pMAT436 | pBJ114, contains 2594 bp gene fragment of *pilA* region with *pilA* gene from 941-1603 bp | This study |
| pMAT473 | pMAT436, construct for endogenous *pilA*-R30Q mutation | This study |
| pMAT474 | pMAT436, construct for endogenous *pilA*-R30A mutation | This study |
| pMAT476 | pMAT436, construct for endogenous *pilA*-K37Q mutation | This study |
| pMAT477 | pMAT436, construct for endogenous *pilA*-K37A mutation | This study |
| pMAT479 | pMAT436, construct for endogenous *pilA*- E53Q mutation | This study |
| pMAT480 | pMAT436, construct for endogenous *pilA*- E53A mutation | This study |
| pMAT485 | pMAT436, construct for endogenous *pilA*-D55N mutation | This study |
| pMAT486 | pMAT436, construct for endogenous *pilA*-D55A mutation | This study |
| pMAT488 | pMAT436, construct for endogenous *pilA*-R73Q mutation | This study |
| pMAT489 | pMAT436, construct for endogenous *pilA*-R73A mutation | This study |
| pMAT491 | pMAT436, construct for endogenous *pilA*-R109Q mutation | This study |
| pMAT492 | pMAT436, construct for endogenous *pilA*-R109A mutation | This study |
| pMAT494 | pMAT436, construct for endogenous *pilA*-K48Q mutation | This study |
| pMAT495 | pMAT436, construct for endogenous *pilA*-K48A mutation | This study |
| pMAT497 | pMAT436, construct for endogenous *pilA*-E69Q mutation | This study |
| pMAT498 | pMAT436, construct for endogenous *pilA*-E69A mutation | This study |
| pMAT528 | pMAT436, construct for endogenous *pilA*-R70Q mutation | This study |
| pMAT505 | pMAT436, construct for endogenous *pilA*-R70A mutation | This study |

**Supplementary Table 5. Oligonucleotides used in this work.**

| **Name** | **Sequence**[1] |
|---|---|
| *pilA*-up EcoRI+ | **GCGCGAATTC**CACTGGCGCGACCACCGAC |
| *pilA*-down HindIII- | **GCGCAAGCTT**GAACTGAATGCCACCCGCC |
| *pilA*-E2 | TTGAACGAGGGGACGCTGGA |
| *pilA*-F3 | TGGCCTGGGGCAATCTCAAG |
| *pilA*-G | CCTGGCCGCCATCGCCATCC |
| *pilA*-H | CGATCACCCAGTCATCGAAG |
| *pilA*-stop- | TTACTGGGCCGCGCCGTC |
| *pilA*-start+ | ATGCGCGTCTCGCGATTC |
| M13 rev | GAGCGGATAACAATTTCACACAGG |
| M13 forw | AGGGTTTTCCCAGTCACGACGTT |
| pilA-R42(30)Q+ | TTCATCAAGTTCCAGGCCCAGTCGAAGCAGTCCGAGGCG |
| pilA-R42(30)Q- | CGCCTCGGACTGCTTCGACTGGGCCTGGAACTTGATGAA |
| pilA-R42(30)A+ | TTCATCAAGTTCCAGGCCGCCTCGAAGCAGTCCGAGGCG |
| pilA-R42(30)A- | CGCCTCGGACTGCTTCGAGGCGGCCTGGAACTTGATGAA |
| pilA-K49(37)Q+ | TCGAAGCAGTCCGAGGCGCAGACGAACCTCAAGGCGCTG |
| pilA-K49(37)Q- | CAGCGCCTTGAGGTTCGTCTGCGCCTCGGACTGCTTCGA |
| pilA-K49(37)A+ | TCGAAGCAGTCCGAGGCGGCCACGAACCTCAAGGCGCTG |
| pilA-K49(37)A- | CAGCGCCTTGAGGTTCGTGGCCGCCTCGGACTGCTTCGA |
| pilA-E65(53)Q+ | CAGAAGTCGTTCTTCTCCCAGAAGGACCGTTACTCCGAC |
| pilA-E65(53)Q- | GTCGGAGTAACGGTCCTTCTGGGAGAAGAACGACTTCTG |
| pilA-E65(53)A+ | CAGAAGTCGTTCTTCTCCGCCAAGGACCGTTACTCCGAC |
| pilA-E65(53)A- | GTCGGAGTAACGGTCCTTGGCGGAGAAGAACGACTTCTG |
| pilA-D67(55)N+ | TCGTTCTTCTCCGAGAAGAACCGTTACTCCGACTTCGCC |
| pilA-D67(55)N- | GGCGAAGTCGGAGTAACGGTTCTTCTCGGAGAAGAACGA |
| pilA-D67(55)A+ | TCGTTCTTCTCCGAGAAGGCGCGTTACTCCGACTTCGCC |

| | |
|---|---|
| pilA-D67(55)A- | GGCGAAGTCGGAGTAACGCGCCTTCTCGGAGAAGAACGA |
| pilA-R85(73)Q+ | GCGCCGGAGCGCGGCAACCAGTACGGCTACCGTGTGTCC |
| pilA-R85(73)Q- | GGACACACGGTAGCCGTACTGGTTGCCGCGCTCCGGCGC |
| pilA-R85(73)A+ | GCGCCGGAGCGCGGCAACGCGTACGGCTACCGTGTGTCC |
| pilA-R85(73)A- | GGACACACGGTAGCCGTACGCGTTGCCGCGCTCCGGCGC |
| pilA R121(109)Q+ | ATCTCCAACGACTCGTTCCAGTTCGGTGCCAACAGCGCC |
| pilA R121(109)Q- | GGCGCTGTTGGCACCGAACTGGAACGAGTCGTTGGAGAT |
| pilA R121(109)A+ | ATCTCCAACGACTCGTTCGCGTTCGGTGCCAACAGCGCC |
| pilA R121(109)A- | GGCGCTGTTGGCACCGAACGCGAACGAGTCGTTGGAGAT |
| pilA K60(48)Q+ | GCGCTGTACACCGCGCAGCAGTCGTTCTTCTCCGAGAAG |
| pilA K60(48)Q- | CTTCTCGGAGAAGAACGACTGCTGCGCGGTGTACAGCGC |
| pilA K60(48)A+ | GCGCTGTACACCGCGCAGGCGTCGTTCTTCTCCGAGAAG |
| pilA K60(48)A- | CTTCTCGGAGAAGAACGACGCCTGCGCGGTGTACAGCGC |
| pilA-E81(69)Q+ | GAAATCGGCTTCGCGCCGCAGCGCGGCAACCGTTACGGC |
| pilA-E81(69)Q- | GCCGTAACGGTTGCCGCGCTGCGGCGCGAAGCCGATTTC |
| pilA-E81(69)A+ | GAAATCGGCTTCGCGCCGGCGCGCGGCAACCGTTACGGC |
| pilA-E81(69)A- | GCCGTAACGGTTGCCGCGCGCCGGCGCGAAGCCGATTTC |
| pilA-R82(70)Q+ | ATCGGCTTCGCGCCGGAGCAGGGCAACCGTTACGGCTAC |
| pilA-R82(70)Q- | GTAGCCGTAACGGTTGCCCTGCTCCGGCGCGAAGCCGAT |
| pilA-R82(70)A+ | ATCGGCTTCGCGCCGGAGGCGGGCAACCGTTACGGCTAC |
| pilA-R82(70)A- | GTAGCCGTAACGGTTGCCCGCCTCCGGCGCGAAGCCGAT |

[1] Sequences added for cloning purposes are indicated in bold. Restriction sites are underlined.

## Supplementary References


1. K. F. Aoki, M. Kanehisa, Using the KEGG database resource. *Curr. Protoc. Bioinformatics* **11**, 1.12.11-11.12.54 (2005).

2. W. Li, A. Godzik, Cd-hit: a fast program for clustering and comparing large sets of protein or nucleotide sequences. *Bioinformatics* **22**, 1658-1659 (2006).

3. F. Teufel *et al.*, SignalP 6.0 predicts all five types of signal peptides using protein language models. *Nat Biotechnol* **40**, 1023-1025 (2022).

4. J. Pei, M. Tang, N. V. Grishin, PROMALS3D web server for accurate multiple protein sequence and structure alignments. *Nucleic Acids Res* **36**, W30-34 (2008).

5. C. Notredame, D. G. Higgins, J. Heringa, T-Coffee: A novel method for fast and accurate multiple sequence alignment. *J. Mol. Biol.* **302**, 205-217 (2000).

6. J. D. Thompson, D. G. Higgins, T. J. Gibson, CLUSTAL W: improving the sensitivity of progressive multiple sequence alignment through sequence weighting, position-specific gap penalties and weight matrix choice. *Nucleic Acids Res.* **22**, 4673-4680 (1994).

7. W. Cai, J. Pei, N. V. Grishin, Reconstruction of ancestral protein sequences and its applications. *BMC Evol Biol* **4**, 33 (2004).

8. F. Gabler *et al.*, Protein sequence analysis using the MPI Bioinformatics Toolkit. *Curr Protoc Bioinformatics* **72**, e108 (2020).

9. I. Letunic, P. Bork, Interactive Tree Of Life (iTOL): an online tool for phylogenetic tree display and annotation. *Bioinformatics* **23**, 127-128 (2007).

10. I. Letunic, S. Khedkar, P. Bork, SMART: recent updates, new developments and status in 2020. *Nucleic Acids Res* **49**, D458-D460 (2021).

11. R. Evans *et al.*, Protein complex prediction with AlphaFold-Multimer. *bioRxiv* 10.1101/2021.1110.1104.463034., 10.1101/2021.1110.1104.463034. (2022).

12. J. Jumper *et al.*, Highly accurate protein structure prediction with AlphaFold. *Nature* **596**, 583-589 (2021).

13. M. Mirdita *et al.*, ColabFold: making protein folding accessible to all. *Nat Methods* **19**, 679-682 (2022).

14. J. Schwabe, M. Pérez-Burgos, M. Herfurth, T. Glatter, L. Søgaard-Andersen, Evidence for a widespread third system for bacterial polysaccharide export across the outer membrane comprising a composite OPX/beta-barrel translocon. *mBio* **13**, e0203222 (2022).

15. A. Treuner-Lange *et al.*, PilY1 and minor pilins form a complex priming the type IVa pilus in *Myxococcus xanthus*. *Nat Commun* **11**, 5054 (2020).

16. J. L. Hutter, J. Bechhoefer, Calibration of atomic-force microscope tips. *Review of Scientific Instruments* **64**, 1868-1873 (1993).

17. D. Kaiser, Social gliding is correlated with the presence of pili in *Myxococcus xanthus*. *Proc. Natl. Acad. Sci. USA* **76**, 5952-5956 (1979).

18. S. S. Wu, D. Kaiser, Regulation of expression of the *pilA* gene in *Myxococcus xanthus*. *J Bacteriol* **179**, 7748-7758 (1997).

19. T. J. Collins, ImageJ for microscopy. *Biotechniques* **43**, 25-30 (2007).

20. F. Wang *et al.*, Cryoelectron microscopy reconstructions of the *Pseudomonas aeruginosa* and *Neisseria gonorrhoeae* type IV pili at sub-nanometer resolution. *Structure* **25**, 1423-1435 e1424 (2017).

21. S. Kolappan *et al.*, Structure of the *Neisseria meningitidis* type IV pilus. *Nat Commun* **7**, 13015 (2016).

22. A. Neuhaus *et al.*, Cryo-electron microscopy reveals two distinct type IV pili assembled by the same bacterium. *Nat Commun* **11**, 2231 (2020).

23. B. Bardiaux *et al.*, Structure and assembly of the enterohemorrhagic *Escherichia coli* type 4 pilus. *Structure* **27**, 1082-1093 e1085 (2019).

24. Y. Gu *et al.*, Structure of *Geobacter* pili reveals secretory rather than nanowire behaviour. *Nature* **597**, 430-434 (2021).

25. R. Anger *et al.*, Structure of a heteropolymeric type 4 pilus from a monoderm bacterium. *Nat Commun* **14**, 7143 (2023).

26. S. S. Wu, J. Wu, D. Kaiser, The *Myxococcus xanthus pilT* locus is required for social gliding motility although pili are still produced. *Mol. Microbiol.* **23**, 109-121 (1997).

27. B. Julien, A. D. Kaiser, A. Garza, Spatial control of cell differentiation in *Myxococcus xanthus*. *Proc. Natl. Acad. Sci. USA* **97**, 9098-9103 (2000).
